# Clinical Correlates of Poor Insight in Obsessive–Compulsive Disorder: An Integrative Cross-Sectional Analysis

**DOI:** 10.3390/jcm15187289

**Published:** 2026-09-19

**Authors:** Maciej Żerdziński, Marcin Burdzik, Roksana Żmuda, Paweł Dębski, Agnieszka Witkowska-Berek, Marek Krzystanek

**Affiliations:** 1 Department of Psychiatry and Sexology, Faculty of Medicine, Academy of Silesia, 43 Rolna Street, 40-555 Katowice, Poland; 2 Psychiatric Department No. 2, Dr. Krzysztof Czuma’s Psychiatric Center, 27 Korczaka Street, 40-340 Katowice, Poland; roksana.zmuda92@gmail.com (R.Ż.); witkowskaa.aga3@gmail.com (A.W.-B.); 3 Faculty of Law and Administration, University of Silesia in Katowice, 11b Bankowa Street, 40-007 Katowice, Poland; 4 Institute of Psychology, Faculty of Social Sciences and Humanities, Humanitas University in Sosnowiec, 43 Kilińskiego Street, 41-200 Sosnowiec, Poland; pdebski@sum.edu.pl; 5 Department of Psychiatry, Faculty of Medical Sciences in Zabrze, Medical University of Silesia in Katowice, 49 Pyskowicka Street, 42-612 Tarnowskie Góry, Poland; 6 Department of Clinical and Community Psychiatry and Psychology, Collegium Medicum, WSB University, 1C Cieplaka Street, 41-300 Dąbrowa Górnicza, Poland; marek.krzystanek@wsb.edu.pl

**Keywords:** obsessive–compulsive disorder, poor insight, insight assessment, Brown Assessment of Beliefs Scale, obsessive-compulsive personality disorder, hoarding

## Abstract

**Background/Objectives**: Poor insight is a clinically relevant feature of obsessive–compulsive disorder (OCD), yet its broader clinical correlates remain incompletely understood. We aimed to characterize the clinical correlates of insight in adults with OCD using complementary categorical and dimensional approaches. **Methods**: In this single-center cross-sectional study, 80 adults with OCD were assessed using the Brown Assessment of Beliefs Scale (BABS) total score and BABS item 6. Insight was examined categorically and dimensionally. FDR-corrected Spearman screening was followed by complementary multivariable models and exploratory graphical-lasso partial-correlation networks and relative-importance analyses. **Results**: Poor-to-absent insight was present in 20 participants (25.0%). In between-group comparisons, the largest differences involved OCD severity, OCPD trait count, and hoarding. In the fully adjusted model for BABS total, OCD severity was the only independently significant correlate (β = 0.339; 95% CI 0.051–0.628; *p* = 0.021). Exploratory conditional-network analysis likewise identified OCD severity as the strongest conditional correlate of BABS total (partial r = 0.251). For BABS item 6, older age retained an adjusted association, while higher YMRS scores were associated with the upper cumulative thresholds; these threshold-specific findings were based on small subgroups and should be interpreted as exploratory. **Conclusions**: Poor-to-absent insight was present in one quarter of this treatment-experienced clinical sample. OCD severity emerged as the most consistent correlate of poorer global insight, whereas BABS item 6 showed a partly different pattern of associations. Categorical and dimensional assessment provided complementary information, but BABS item 6 is part of the BABS total score and the observed differences should not be interpreted as evidence of independent insight constructs. Clinical assessment should therefore consider both the level of insight itself and the broader psychopathological context.

## 1. Introduction

### 1.1. Obsessive–Compulsive Disorder

Obsessive–compulsive disorder (OCD), with an estimated global lifetime prevalence of approximately 2.3–3.2%, depending on diagnostic criteria [1], is characterized by obsessions and compulsions, or both [2]. Obsessions are recurrent and persistent thoughts, images, or urges experienced as intrusive and unwanted, typically accompanied by distress and by attempts to ignore, suppress, or neutralize them. Compulsions are repetitive behaviors or mental acts that individuals feel driven to perform in response to an obsession or according to rigidly applied rules, usually to reduce distress or prevent a feared event [2,3]. In addition to these defining symptoms, patients may experience pervasive feelings of incompleteness, “not-just-right” experiences, and other sensory phenomena that precede or accompany compulsive behavior [4,5,6]. Despite its characteristic clinical presentation, OCD remains frequently underrecognized [7] and is associated with a markedly prolonged duration of untreated illness, commonly ranging from 7 to 13 years [8,9,10]. In individual cases, the interval between symptom onset and treatment initiation may extend over several decades, placing OCD among the mental disorders associated with the longest delays in receiving appropriate care [11]. Delayed help-seeking has been linked to limited recognition of symptoms, shame, stigma, and reluctance to disclose distressing obsessive content [12]. Psychiatric comorbidity, particularly mood disorders, is common in OCD and has been associated with a more severe clinical course and less favorable outcomes [13,14,15]. Cognitive and affective characteristics, including impulsivity [16], anger, and aggression [17,18,19], may additionally contribute to the marked clinical heterogeneity of OCD. Finally, OCD is associated with persistent reductions in quality of life across social, occupational, and interpersonal domains [20,21,22].

### 1.2. Insight

Insight is commonly understood as the capacity to recognize psychopathological experiences, attribute them to a mental disorder, and acknowledge their clinical implications, including the need for treatment. However, no universally accepted definition exists, and differences in its conceptualization and measurement limit comparability across studies and clinical assessments [23,24,25]. Early theoretical accounts already emphasized that insight is not a unitary construct. Lerner (1966) examined the relationships among insight, judgment, and functioning while cautioning against unsupported assumptions regarding their contribution to psychiatric disability [26]. Brady (1967) distinguished intellectual insight—the cognitive recognition of illness without a necessary corresponding change in behavior—from emotional insight, understood as a more affectively integrated form of understanding capable of facilitating psychological change [27]. Hatcher (1973), in turn, emphasized self-observation as a process through which insight may develop [28]. A particularly influential framework was proposed by David (1990), who described three central components of insight: recognition of having a mental disorder, attribution of symptoms to that disorder, and acknowledgment of the need for treatment [29]. Although originally formulated in relation to psychotic disorders, this framework was subsequently applied to other psychiatric conditions, including OCD [25].

### 1.3. Insight in OCD

In the ICD-10 research diagnostic criteria, OCD was described predominantly in terms of a presentation characterized by preserved insight and ego-dystonicity: obsessive thoughts and compulsive acts were recognized as originating in the individual’s own mind, at least one symptom was regarded as excessive or unreasonable, and resistance was typically present [30]. This formulation provided limited scope for representing the full range of insight observed in OCD. More recent accounts conceptualize insight in OCD in terms of the perceived plausibility or reasonableness of obsession-related beliefs and the extent to which obsessions and compulsions are recognized as symptoms of the disorder [31]. The sensory properties and subjective vividness of obsessive experiences may make the associated compulsions more compelling and difficult to resist [32]. Poor insight in OCD cannot, however, be reduced to deficient rational self-appraisal or attributed exclusively to cognitive limitations. Marková et al. (2009) conceptualized insight as a form of self-knowledge and argued that it should be understood as a mental state rather than merely as a symptom or symptom dimension [33]. Consistent with this broader account, de Avila et al. (2019) situated insight in OCD within a wider process of self-appraisal involving decision-making, anticipation of consequences, and the perception of one’s capacity to act [34]. Neuropsychological findings further suggest that poorer insight may be associated with weaker performance in executive domains, including cognitive flexibility, set shifting, conflict resolution, and response inhibition, as well as in verbal learning and memory [35,36]. Insight in OCD has also been associated with affective symptoms, particularly depressive severity [15], and with social-cognitive performance, especially theory-of-mind reasoning [37].

The clinical variability of insight is formally acknowledged in contemporary diagnostic classifications. The DSM-5 distinguishes OCD with good or fair insight, poor insight, and absent insight/delusional beliefs [2], whereas the ICD-11 applies two broader specifiers: fair-to-good insight and poor-to-absent insight [3]. This dimensional approach is consistent with evidence that obsessive–compulsive symptoms vary in the extent to which they are experienced as ego-dystonic or ego-syntonic, thereby influencing their recognition as excessive, unreasonable, or pathological [38,39]. Studies employing different definitions, assessment instruments, and categorical thresholds have produced widely divergent estimates of poor insight. Marazziti et al. (2002) classified 85% of patients as having excellent, good, or sufficient insight, whereas poor and absent insight were identified in 10% and 5%, respectively [40]. Matsunaga et al. (2002) reported excellent, good, or fair insight in 64% of the sample, poor insight in 29%, and absent insight in 7% [41]. Alonso et al. (2008) identified poor insight in 29.5% of participants [42], while Catapano et al. (2010) found good insight in 78.3% and poor insight in 21.7% in a three-year prospective study [43]. More recent estimates remain similarly variable: Guillén-Font et al. (2021) reported poor-to-absent insight in 26.2% of cases [23], whereas Grover et al. (2021) found poor insight in only 11.2% and good or fair insight in the remaining 88.8% [44]. Guillén-Font et al. (2021) further associated poorer insight with lower overall functioning, unemployment, and a less favorable course of illness, whereas fair insight was more frequently observed among patients with an episodic course and higher educational attainment [23]. A family history of OCD may also occur more frequently among patients with poor insight [45], while higher rates of schizophrenia-spectrum disorders have been reported among their first-degree relatives [43]. Insight also appears to vary with the phenomenological expression of obsessive–compulsive symptoms (OCS), although the pattern of associations has been inconsistent. Poorer insight has been associated with reduced resistance to obsessions and compulsions, poorer control over obsessions, and a greater number of OCS, particularly symmetry-related and hoarding symptoms [42,46]. Other findings have been less uniform: contamination obsessions and cleaning compulsions were more frequent among patients with good insight in one study, whereas no differences were observed for aggressive/checking, sexual/religious, symmetry/ordering, or hoarding symptoms [42]. In another sample, the association between insight and resistance or control over symptoms was not confirmed [40].

In dimension-specific analyses, depressive severity and comorbid generalized anxiety disorder were associated with poorer insight into aggressive/checking symptoms, earlier OCD onset with poorer insight into sexual/religious and symmetry symptoms, and later onset with poorer insight into hoarding obsessions [47]. Psychiatric comorbidity and personality-related characteristics have also been linked to insight in OCD. Greater depressive symptom severity and a higher prevalence of personality disorders, particularly schizotypal personality disorder, have been reported among patients with poor insight [42,43]. Furthermore, comorbid anankastic personality was associated with a broader clinical profile characterized by poorer insight, higher OCD severity, more extensive psychiatric comorbidity, and poorer functioning [48]. Findings regarding treatment outcome have been less consistent. In a three-year prospective study, patients with poor insight were less likely to achieve remission, underwent more therapeutic trials, and more frequently received antipsychotic augmentation [43], whereas other studies found no significant effect of baseline insight on pharmacological response [42,49].

Given the conceptual complexity of insight in OCD and the variability of its clinical expression, an important gap remains in the existing literature. Most previous studies have examined associations between individual clinical characteristics and the level of insight, whereas considerably less attention has been paid to how these characteristics are interrelated within the broader clinical context in which insight is expressed. It also remains unclear whether the pattern of associations observed for BABS total is reproduced when BABS item 6 is considered separately, while recognizing that item 6 contributes to the total score.

## 2. Objectives

The primary objective of this study was to characterize insight in a clinical sample of adults with obsessive–compulsive disorder, considering both its dimensional distribution and the frequency of poor-to-absent insight. The secondary objectives were:

1. To compare the sociodemographic, clinical, and phenomenological characteristics of patients with fair-to-good and poor-to-absent insight.
2. To examine, across the full sample, the dimensional associations of BABS total and BABS item 6 with sociodemographic, clinical, affective, personality, impulsivity, aggression, and symptom-dimensional variables.
3. To determine which clinical correlates showed distinct associations with the insight outcomes after multivariable adjustment and to explore their broader conditional interrelationships and relative contributions.

## 3. Materials and Methods

### 3.1. Participants and Clinical Assessment

This cross-sectional study included 80 adult patients with a primary diagnosis of OCD, recruited between December 2022 and March 2025 from the inpatient and outpatient services of the Dr. Krzysztof Czuma Psychiatric Center in Katowice, Poland. Potentially eligible patients were identified among patients with a known primary diagnosis of OCD treated at the Center and were approached consecutively. During the recruitment period, 88 eligible patients were invited to participate; eight declined because they did not wish to complete the study assessment battery, and 80 were enrolled, corresponding to a participation rate of 90.9%. Of the 80 enrolled participants, 6 were recruited from inpatient services and 74 from outpatient services. Participants were eligible if they were aged 18 years or older, had previously received treatment for OCD, and had a primary DSM-5 diagnosis of OCD independently confirmed by at least two experienced clinicians. Eligibility also required the capacity to provide informed consent and complete the study assessments. No additional study-specific exclusion criteria were applied. At the time of assessment, no participant was experiencing a depressive or manic episode with psychotic features, and none had a comorbid schizophrenia-spectrum disorder. All clinician-rated scales used in the study were independently administered by two experienced clinicians. Discrepant ratings were reviewed jointly, and final scores were established by consensus. Sex was recorded as female or male from the clinical records. All participants received a detailed explanation of the study aims and procedures and provided written informed consent. The study procedures were approved by the Bioethics Committee of the Medical University of Silesia in Katowice (approval number PCN/CBN/0052/KB/235/22; 13 December 2022).

The severity of obsessive–compulsive symptoms was assessed using the Yale–Brown Obsessive–Compulsive Scale (Y-BOCS), a clinician-rated instrument yielding a total score ranging from 0 to 40 and separate obsession and compulsion subscale scores ranging from 0 to 20 each [50]. Higher scores indicate greater symptom severity. Based on the total score, OCD severity was classified as mild (8–15), moderate (16–23), severe (24–31), or extreme (32–40). Symptoms recorded on the Y-BOCS Symptom Checklist were additionally grouped into five checklist-derived dimensions: contamination/cleaning, taboo thoughts, doubts/checking, symmetry/ordering, and hoarding [51].

Insight was assessed using the Brown Assessment of Beliefs Scale (BABS), a seven-item clinician-rated semistructured instrument evaluating insight during the preceding 7 days [52]. Items 1–6 contribute to the total score, which ranges from 0 to 24, whereas item 7 is scored separately and is not included in the total. Higher total scores indicate poorer insight. Insight was analyzed both dimensionally and categorically. For categorical analyses, participants were classified as having fair-to-good insight (BABS total ≤12) or poor-to-absent insight (BABS total >12). The >12 threshold is consistent with published BABS categorizations in which scores of 8–12 indicate fair insight and scores above 12 fall within poor or delusional categories depending on the total score and conviction rating; the same >12 threshold has also been used to define poor insight in OCD samples [35]. BABS item 6 (range, 0–4) was additionally analyzed as a secondary insight-related outcome.

Depressive symptom severity was assessed using the 17-item Hamilton Depression Rating Scale (HDRS-17), a clinician-rated instrument widely used to quantify depressive symptoms, with individual items rated on either 3-point or 5-point scales [53]. Manic symptom severity was assessed using the Young Mania Rating Scale (YMRS), an 11-item clinician-rated instrument in which seven items are scored from 0 to 4 and four items from 0 to 8, yielding a total score ranging from 0 to 60 [54]. Both measures were analyzed dimensionally, with higher scores indicating greater symptom severity.

Aggression was assessed using the Buss–Perry Aggression Questionnaire (BPAQ), a 29-item self-report instrument measuring total aggression and four related dimensions: physical aggression, verbal aggression, anger, and hostility. Items are rated on a 5-point Likert scale, yielding a total score ranging from 29 to 145 [55]. Impulsivity was assessed using the Barratt Impulsiveness Scale, version 11 (BIS-11), a 30-item self-report instrument measuring overall impulsivity and its cognitive/attentional, motor, and non-planning dimensions, with a total score ranging from 30 to 120 [56]. Higher scores indicate greater aggression and impulsivity, respectively.

The psychiatric comorbidity count was calculated as the number of the following categories present: major depressive disorder, anxiety disorders, bipolar disorder, and alcohol dependence/abuse; the observed range was 0–3. The count was used as an aggregate indicator of psychiatric complexity rather than as a measure of the clinical impact of individual comorbid disorders. Each diagnostic category was verified separately on the basis of clinician-administered interviews and available psychiatric records and documented using a structured clinician-completed verification form. OCPD traits were assessed separately from psychiatric comorbidity by clinician-led evaluation with reference to the eight DSM-5 criteria [2]. Each criterion was rated as present or absent and summed to yield the dimensional OCPD trait count (range, 0–8). All diagnostic conclusions and OCPD trait ratings were reviewed by two experienced clinicians. The structured verification form served as an aid to clinical assessment rather than as a stand-alone structured personality-disorder interview; an English translation is provided in Supplementary Materials S2.

Clinical variables included illness duration, treatment delay, treatment duration, and illness course. Illness duration was calculated as the interval between OCD symptom onset and study assessment; treatment delay as the interval between symptom onset and initiation of OCD treatment; and treatment duration as the interval between treatment initiation and study assessment. Illness course was classified as episodic when at least one remission lasting ≥12 months had occurred and as chronic when no such remission had occurred.

### 3.2. Statistical Analysis

Distributional normality was assessed using the Shapiro–Wilk test. Normally distributed continuous variables were presented as means with standard deviations, non-normally distributed continuous and ordinal variables as medians with interquartile ranges, and categorical variables as numbers and percentages. Quartiles in the between-group analyses were calculated using the midpoint method. Patients with fair-to-good and poor-to-absent insight were compared using the independent-samples Student’s *t* test, Mann–Whitney *U* test, Pearson’s χ^2^ test, or Fisher’s exact test, as appropriate. Effect sizes were reported as Cohen’s d, r = z/√N, or Cramér’s V. There were no missing data for the variables included in the analyses. No formal a priori sample-size calculation was performed; the sample size was determined by consecutive recruitment during the study period. Given the sample size and the multistage analytical strategy, the network and relative-importance analyses were considered exploratory.

Whole-sample dimensional associations were examined separately for BABS total and BABS item 6 using Spearman rank correlations with 30 sociodemographic, clinical, affective, personality, impulsivity, aggression, and checklist-derived symptom variables. The Benjamini–Hochberg procedure was applied separately for each outcome, controlling the false discovery rate at 5%. Variables retaining q < 0.05 were used to define the subsequent network candidate set. Complete screening results and the full correlation matrix are provided in Supplementary Table S1, Supplementary Figure S1, and Supplementary Data S1.

To distinguish conditional associations from pairwise relationships shared with other clinical characteristics, the retained correlates were examined using separate graphical-lasso partial-correlation networks for BABS total and BABS item 6. The candidate set comprised the union of variables retaining an FDR-significant association with either outcome. Because Y-BOCS total, obsession, and compulsion scores are structurally overlapping measures, only Y-BOCS total was retained for the network representation. The resulting common candidate set comprised age, employment status, illness duration, episodic course, Y-BOCS total, psychiatric comorbidity count, OCPD trait count, hoarding, and YMRS total. Variables underwent rank-based Gaussian transformation, and the regularization penalty was selected by five-fold cross-validation. Network edges represented partial correlations after accounting for all remaining nodes, and their stability was assessed using 1000 nonparametric bootstrap resamples. Detailed procedures and complete estimates are reported in the Supplementary Methods, Supplementary Table S2, and Supplementary Data S2.

As the final integrative stage, the exact seven-variable set was defined after dimensional screening from the retained candidate variables, with structurally overlapping measures excluded and clinically interpretable, nonredundant variables retained; no stepwise selection procedure was used. Relative-importance analysis then quantified the contribution of Y-BOCS total, OCPD trait count, YMRS total, age, illness duration, psychiatric comorbidity count, and hoarding to model fit. Employment status and episodic course remained part of the broader network representation and were evaluated in extended sensitivity models rather than in the primary seven-variable analyses. For BABS total, *R*^2^ was decomposed using the LMG/Shapley method. For BABS item 6, violation of the proportional-odds assumption for YMRS total led to decomposition of McFadden pseudo-*R*^2^ from separate binary logistic models for the cumulative contrasts >0 versus 0, >1 versus 0–1, and >2 versus 0–2. Stability of relative-importance estimates and rankings was evaluated using 1000 nonparametric bootstrap resamples. Across the network and relative-importance analyses, bootstrap procedures were used to assess internal stability and were not interpreted as evidence of external reproducibility.

Complementary fully adjusted analyses included the same seven clinical variables entered simultaneously for each outcome. BABS total was analysed using ordinary least-squares multiple regression with standardized coefficients and HC3 heteroskedasticity-robust standard errors. BABS item 6 was analysed using a partial proportional-odds ordinal logistic model, allowing the YMRS coefficient to vary across cumulative thresholds because the proportional-odds assumption was violated for this variable. Continuous predictors were standardized before multivariable modeling. Prespecified sensitivity analyses examined alternative model specifications and coefficient stability. Core estimates from the complementary fully adjusted models are presented in the Results section. Bootstrap relative-importance results are reported in Supplementary Table S3, whereas complete sensitivity analyses and diagnostic outputs are provided in Supplementary Table S4 and Supplementary Data S2. All statistical tests were two-sided. Statistical significance was set at *p* < 0.05. For the dimensional screening analyses, multiplicity was controlled using the Benjamini–Hochberg procedure with the false discovery rate set at 5%; FDR-adjusted q values < 0.05 were considered statistically significant. Statistical analyses were performed using Statistica 13.3 (TIBCO Software Inc., Palo Alto, CA, USA) and Python 3.13 (Python Software Foundation, Beaverton, OR, USA); figures were prepared using Matplotlib 3.10.8 (Matplotlib Development Team). During manuscript preparation, ChatGPT (GPT-5.6 Sol; OpenAI, San Francisco, CA, USA) was used for editorial and language support, English translation, review of citation structure and sequence, and consistency checking of results reported across the text, tables, and figures. All AI-assisted output was critically reviewed and verified by the first author, who retains full responsibility for the content of the manuscript.

## 4. Results

### 4.1. Sample Characteristics

The study included 80 patients with OCD, including 44 women (55.0%) and 36 men (45.0%), with a mean age of 44.5 ± 12.6 years. Most participants had tertiary education (57.5%), were in a relationship (63.8%), and were occupationally active (61.3%). OCD followed a predominantly chronic course (93.8%), with a mean illness duration of 22.2 ± 11.1 years, a median treatment delay of 10.0 years, and a median treatment duration of 7.0 years. The mean Y-BOCS total score was 22.7 ± 7.1; severe or extreme OCD was present in 50.0% of participants, whereas 18.8% had mild OCD. Overall, 87.5% of participants had at least one assessed psychiatric comorbidity, and 43.8% had two. Detailed sociodemographic and clinical characteristics, including checklist-derived OCD dimensions, are presented in Table 1.

Descriptive statistics for the psychometric measures included in the study, including the BABS total score and BABS item 6, are presented in Table 2.

### 4.2. Clinical Characteristics According to Insight Group

To further characterize the clinical profile associated with insight level, patients were divided into fair-to-good insight (BABS total ≤12) and poor-to-absent insight (BABS total >12) groups. As expected from the grouping criterion, the poor-to-absent insight group had higher BABS total scores than the fair-to-good insight group (16.0 [15.0–17.5] vs. 8.5 [4.0–11.0], *p* < 0.001). BABS item 6 scores were also higher in the poor-to-absent insight group (2.5 [2.0–3.0] vs. 1.0 [0.0–2.0], *p* < 0.001). Compared with patients with fair-to-good insight, those with poor-to-absent insight were older (49.6 ± 13.8 vs. 42.8 ± 11.8 years, *p* = 0.036) and had greater OCD severity, with higher Y-BOCS total (27.7 ± 6.4 vs. 21.0 ± 6.6, *p* < 0.001), obsession (14.5 [12.5–16.0] vs. 12.0 [8.0–13.0], *p* < 0.001), and compulsion scores (13.5 ± 3.6 vs. 10.1 ± 3.5, *p* < 0.001). They also had a higher psychiatric comorbidity count (2.0 [1.5–2.0] vs. 1.0 [1.0–2.0], *p* = 0.035) and a higher OCPD trait count (6.0 [4.0–7.5] vs. 3.0 [2.0–4.0], *p* < 0.001). Illness duration was numerically longer in the poor-to-absent insight group, although the difference was not statistically significant (26.0 ± 13.6 vs. 21.0 ± 10.0 years, *p* = 0.081, d = −0.46). Among the checklist-derived OCD dimensions, hoarding was significantly higher in the poor-to-absent insight group (1.0 [0.5–2.0] vs. 0.0 [0.0–1.0], *p* = 0.003, r = −0.33). Conversely, taboo thoughts were numerically lower, although not statistically significant (1.0 [0.5–2.0] vs. 2.0 [1.0–2.0], *p* = 0.058, r = 0.21). No statistically significant between-group differences were found for sex, education, relationship status, employment, treatment delay, treatment duration, OCD course, HDRS total, YMRS total, impulsivity, aggression, contamination/cleaning, doubts/checking, or symmetry/ordering. Because multiplicity was not controlled in the between-group analyses, findings based on nominal *p* values close to 0.05, particularly those for age and psychiatric comorbidity count, should be interpreted as exploratory. Detailed comparisons are presented in Table 3.

**Figure 1:**
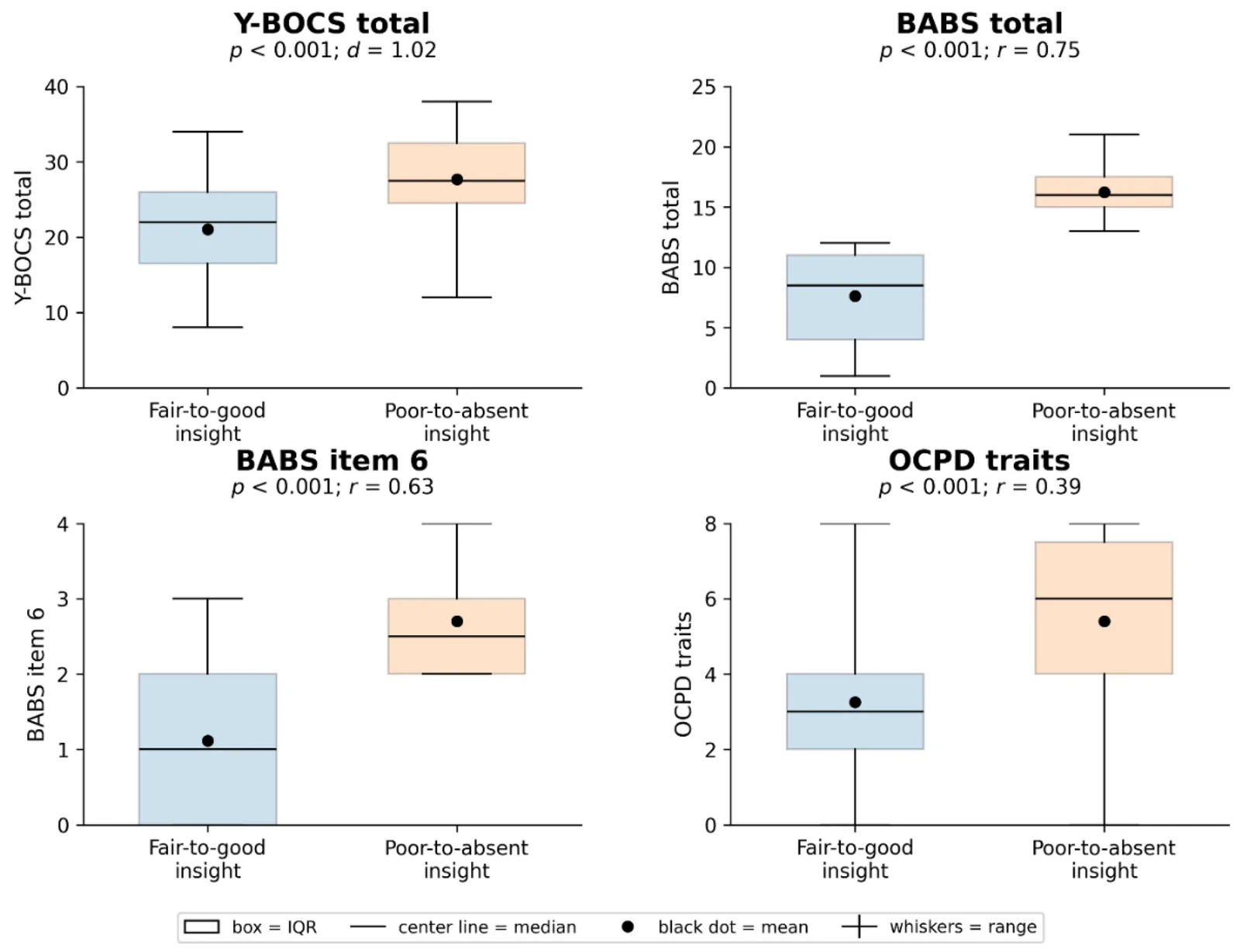
Y-BOCS total score, BABS total score, BABS item 6 score, and OCPD trait count according to insight group (N = 80). Note. d denotes Cohen’s d; r denotes the effect size derived from the Mann–Whitney U test. Abbreviations: BABS, Brown Assessment of Beliefs Scale; OCPD, obsessive–compulsive personality disorder; Y-BOCS, Yale–Brown Obsessive–Compulsive Scale.

Figure 1 illustrates the principal between-group differences in OCD severity, insight measures, and OCPD trait count between patients with fair-to-good and poor-to-absent insight.

### 4.3. Dimensional Associations and Conditional Clinical Networks

To determine whether the clinical differences identified between insight groups extended across the full distribution of insight, subsequent analyses examined insight dimensionally and within conditional clinical networks. After Benjamini–Hochberg correction across 30 candidate variables, 10 variables remained significantly associated with BABS total and 7 with BABS item 6. For BABS total, the strongest pairwise dimensional associations involved Y-BOCS obsessions (r = 0.535; q < 0.001), Y-BOCS total (r = 0.533; q < 0.001), Y-BOCS compulsions (r = 0.483; q < 0.001), OCPD trait count (r = 0.428; q < 0.001), and psychiatric comorbidity count (r = 0.409; q < 0.001). Additional FDR-significant associations were observed with illness duration, hoarding, age, episodic course, and employment status. For BABS item 6, the strongest associations involved Y-BOCS obsessions (r = 0.387; q = 0.010), age (r = 0.369; q = 0.010), Y-BOCS total (r = 0.361; q = 0.010), YMRS total (r = 0.333; q = 0.017), and OCPD trait count (r = 0.329; q = 0.017); illness duration and Y-BOCS compulsions were also retained after FDR correction. Complete screening results are presented in Supplementary Table S1 and Supplementary Figure S1.

**Figure 2:**
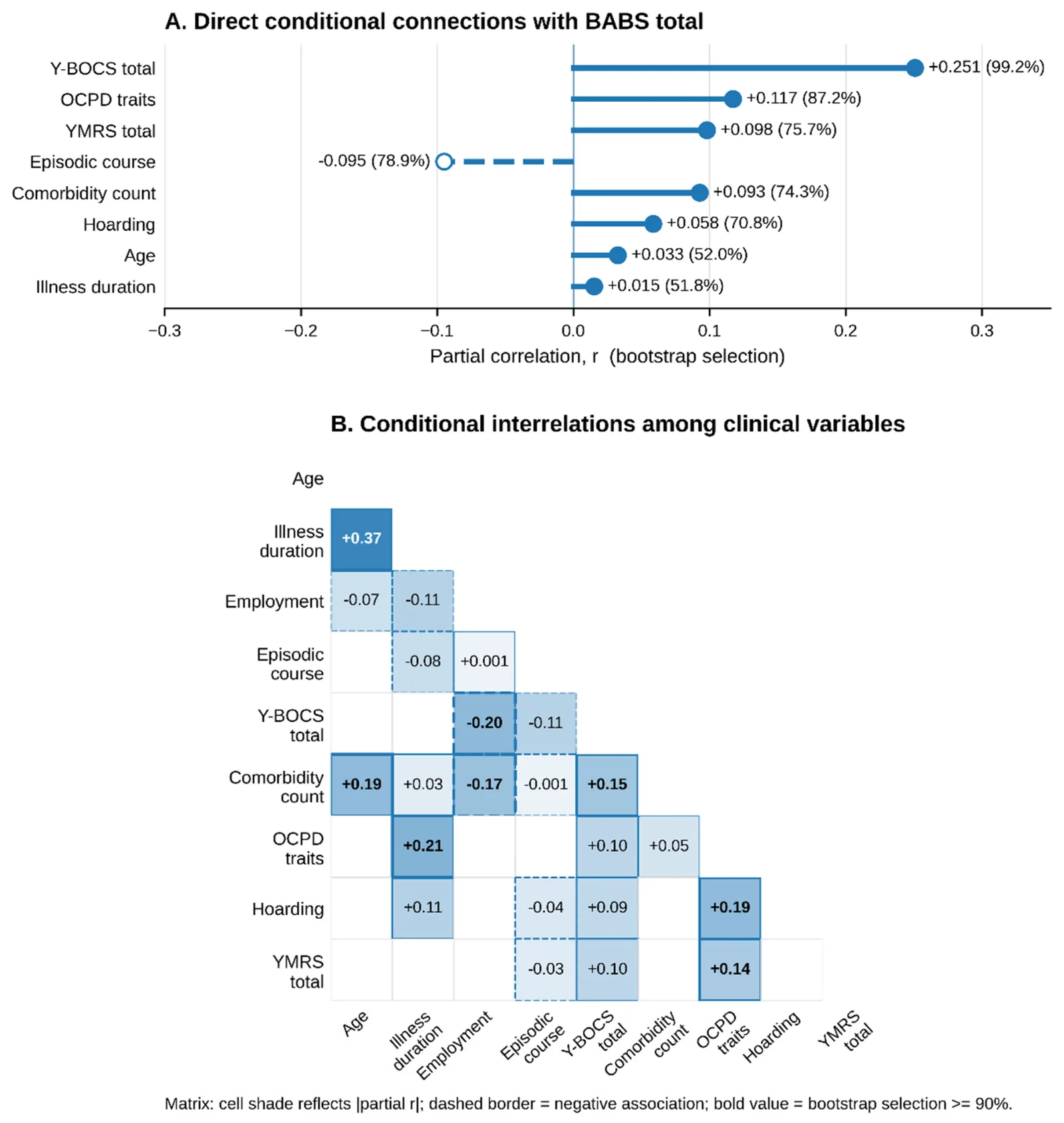
Network of clinical correlates of BABS total (N = 80). Note. Panel (**A**) shows conditional associations with BABS total; percentages indicate bootstrap selection frequencies, defined as the percentage of 1000 bootstrap resamples in which a given edge was retained. Panel (**B**) shows conditional interrelations among clinical variables. An edge represents a non-zero partial correlation retained after graphical-lasso regularization, conditional on all other variables in the network. Cell shading reflects |partial r|; dashed borders indicate negative associations, and bold values indicate bootstrap selection ≥90%. Blank cells indicate no retained edge in the regularized network and should not be interpreted as evidence of absence of a univariate association. Abbreviations: BABS, Brown Assessment of Beliefs Scale; OCPD, obsessive–compulsive personality disorder; Y-BOCS, Yale–Brown Obsessive–Compulsive Scale; YMRS, Young Mania Rating Scale.

Because these dimensional associations do not distinguish direct relationships from those shared with other clinical characteristics, the retained variables were then examined within conditional partial-correlation networks. In the BABS total network, Y-BOCS total showed the strongest direct connection with the outcome (partial r = 0.251), whereas all other direct connections were substantially weaker, as shown in Panel A of Figure 2. The broader pattern of conditional interrelations among the clinical variables is shown in Panel B of Figure 2. The strongest was between age and illness duration (partial r = 0.368), followed by illness duration with OCPD traits (0.215), employment with Y-BOCS total (−0.195), age with psychiatric comorbidity count (0.188), OCPD traits with hoarding (0.188), employment with psychiatric comorbidity count (−0.169), Y-BOCS total with psychiatric comorbidity count (0.155), and OCPD traits with YMRS total (0.139).

**Figure 3:**
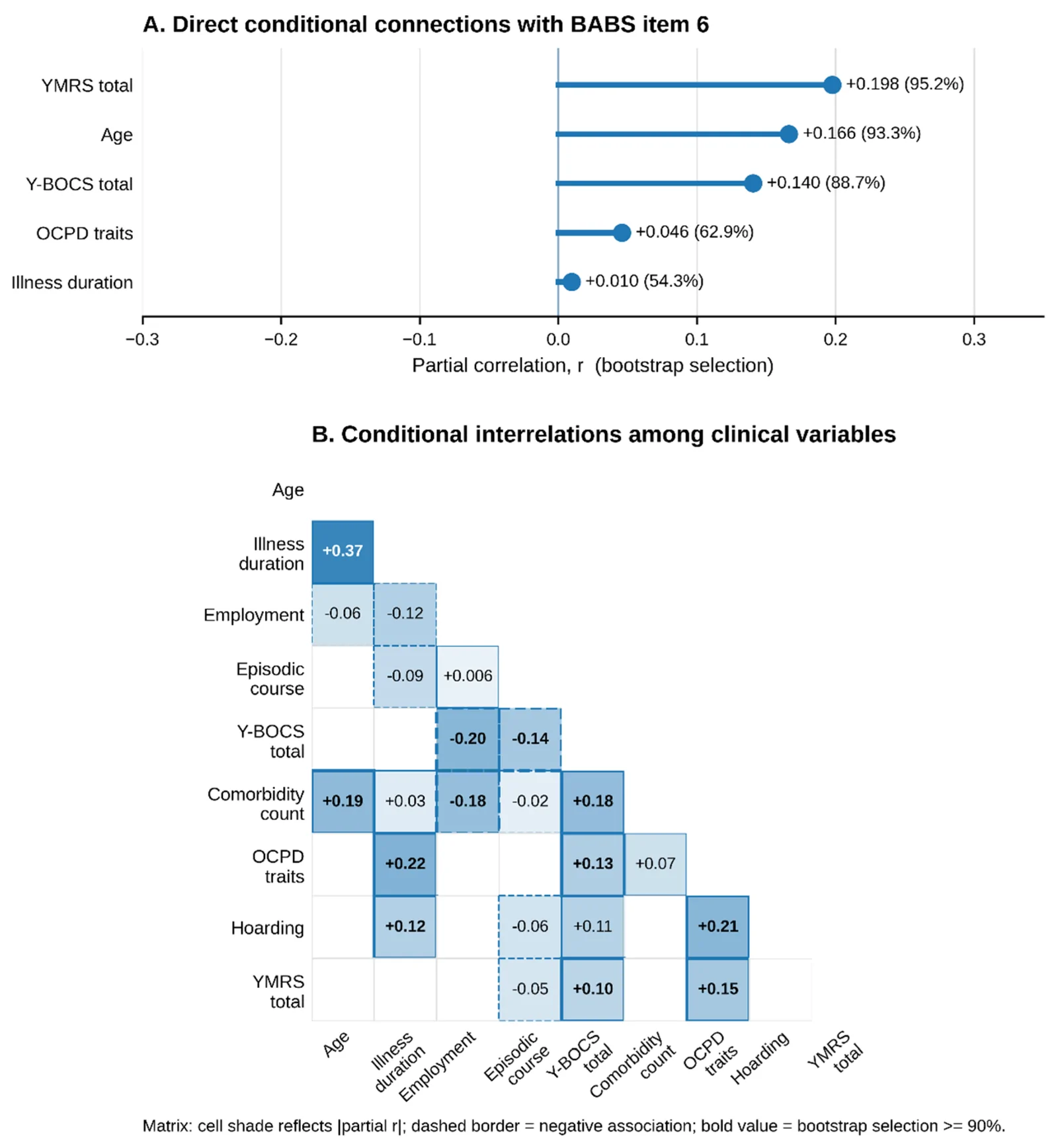
Network of clinical correlates of BABS item 6 (N = 80). Note. Panel (**A**) shows conditional associations with BABS item 6; percentages indicate bootstrap selection frequencies, defined as the percentage of 1000 bootstrap resamples in which a given edge was retained. Panel (**B**) shows conditional interrelations among clinical variables. An edge represents a non-zero partial correlation retained after graphical-lasso regularization, conditional on all other variables in the network. Cell shading reflects |partial r|; dashed borders indicate negative associations, and bold values indicate bootstrap selection ≥90%. Blank cells indicate no retained edge in the regularized network and should not be interpreted as evidence of absence of a univariate association. Abbreviations: BABS, Brown Assessment of Beliefs Scale; OCPD, obsessive–compulsive personality disorder; Y-BOCS, Yale–Brown Obsessive–Compulsive Scale; YMRS, Young Mania Rating Scale.

For BABS item 6, the strongest direct connections with the outcome were observed for YMRS total (partial r = 0.198), age (partial r = 0.166), and Y-BOCS total (partial r = 0.140). Direct connections with OCPD trait count (partial r = 0.046) and illness duration (partial r = 0.010) were weak, whereas psychiatric comorbidity count, hoarding, employment, and episodic course did not retain direct outcome connections, as shown in Panel A of Figure 3. The pattern of conditional interrelations among the clinical variables, shown in Panel B of Figure 3, closely paralleled that observed in the BABS total network. The strongest connection was again between age and illness duration (partial r = 0.368), followed by illness duration with OCPD traits (0.219), OCPD traits with hoarding (0.205), and employment with Y-BOCS total (−0.203). Additional interrelations involved age and psychiatric comorbidity count, Y-BOCS total and psychiatric comorbidity count, employment and psychiatric comorbidity count, and OCPD traits and YMRS total.

Bootstrap analyses supported the stability of the principal direct outcome connections: Y-BOCS total–BABS total was retained in 99.2% of resamples, YMRS total–BABS item 6 in 95.2%, and age–BABS item 6 in 93.3%. Complete edge-level estimates and bootstrap stability measures are provided in Supplementary Table S2.

### 4.4. Relative-Importance Analysis

Relative-importance analysis quantified how the seven clinically interpretable variables contributed to model fit. For BABS total, the seven-variable model explained 40.0% of the observed variance (R^2^ = 0.400). Y-BOCS total accounted for the largest share of the full-model R^2^ (37.2%), followed by YMRS total (18.6%), OCPD trait count (13.6%), psychiatric comorbidity count (11.6%), hoarding (7.1%), illness duration (6.3%), and age (5.6%). Bootstrap rankings supported the predominance of Y-BOCS total, which ranked first in 66.1% of resamples and among the two leading contributors in 86.7% (Supplementary Table S3). For BABS item 6, the relative-importance profile differed across cumulative contrasts. At >0 versus 0, age made the largest contribution (40.3%), followed by illness duration (25.6%). At >1 versus 0–1, OCPD trait count (28.9%) and Y-BOCS total (24.7%) contributed most, whereas at >2 versus 0–2, YMRS total predominated (53.8%), followed by age (20.0%) and Y-BOCS total (11.2%). Bootstrap rankings supported this changing hierarchy across thresholds (Supplementary Table S3). The >3 versus 0–3 contrast was not included in the primary relative-importance analysis because only four participants exceeded that threshold. Accordingly, threshold-specific findings for BABS item 6 should be regarded as exploratory and hypothesis-generating.

### 4.5. Complementary Multivariable Analyses

Complementary fully adjusted analyses examined the same seven clinical variables: Y-BOCS total, OCPD trait count, YMRS total, age, illness duration, psychiatric comorbidity count, and hoarding, entered simultaneously for each insight outcome. For BABS total, the seven-variable model explained 40.0% of the observed variance (R^2^ = 0.400; adjusted R^2^ = 0.342; global *p* < 0.001). Importantly, Y-BOCS total was the only variable retaining a statistically significant adjusted association with BABS total (standardized β = 0.339; 95% CI 0.051–0.628; *p* = 0.021); none of the remaining variables was significant. For BABS item 6, the partial proportional-odds model was also significant (LR χ^2^(10) = 33.968; global *p* < 0.001; McFadden pseudo-R^2^ = 0.151). Older age was associated with higher BABS item 6 categories (OR = 1.975 per 1-SD increase; 95% CI 1.115–3.499; *p* = 0.020). The association with YMRS total was threshold-specific, reaching significance for BABS item 6 >2 versus 0–2 (OR = 3.021; 95% CI 1.452–6.286; *p* = 0.003) and >3 versus 0–3 (OR = 2.622; 95% CI 1.285–5.350; *p* = 0.008), but not at the two lower thresholds. The >3 versus 0–3 estimate was based on only four participants above the threshold and should therefore be regarded as exploratory and interpreted with particular caution. No other variables retained statistically significant adjusted associations. Complete estimates are presented in Table 4; prespecified sensitivity analyses are reported in Supplementary Table S4 and Supplementary Data S2.

## 5. Discussion

Against the background of the literature on insight in OCD reviewed in the Introduction, we identified an important gap in how its clinical correlates have typically been examined. Most previous studies have focused on associations between individual clinical characteristics and the level of insight, whereas their broader interrelationships have received considerably less attention. The present study therefore represents an attempt to address this gap by examining multiple clinical correlates within a common analytical framework. Rather than considering individual correlates in isolation, we sought to characterize their broader interrelationships using complementary categorical, dimensional, multivariable, network, and relative-importance approaches. This integrative perspective was intended to move beyond simple one-to-one associations and to provide a broader context for understanding how variation in insight is situated within the clinical presentation of OCD.

### 5.1. Frequency and Clinical Profile of Poor-to-Absent Insight

Poor-to-absent insight was identified in 20 of 80 patients (25.0%). This proportion closely resembles the 21.7% reported by Catapano et al. (2010) [43] and the 26.2% reported by Guillén-Font et al. (2021) [23], while falling between the lower estimates of Marazziti et al. (2002) [40] and Grover et al. (2021) [44] and the higher rates observed by Matsunaga et al. (2002) [41] and Alonso et al. (2008) [42]. Differences between studies probably reflect variation in sample characteristics, the operationalization of insight, and the categorical thresholds applied. In the present sample, poor-to-absent insight represented a substantial clinical subgroup, consistent with several previous clinical studies. This finding is also compatible with the graded approach adopted in DSM-5 and ICD-11 [2,3] and contrasts with the ICD-10 research diagnostic criteria, in which recognition of obsessive–compulsive symptoms as excessive or unreasonable formed part of the diagnostic formulation [30].

The clearest clinical difference concerned OCD severity. Patients with poor-to-absent insight had higher overall severity than those with fair-to-good insight (27.7 vs. 21.0; d = −1.02; *p* < 0.001), with corresponding differences in both obsessions (14.5 vs. 12.0; r = −0.40; *p* < 0.001) and compulsions (13.5 vs. 10.1; d = −0.97; *p* < 0.001). This finding accords with one of the most consistently reported associations of poor insight in OCD [15,41,42,43,46,57].

Anankastic traits were another prominent feature. Patients with poor-to-absent insight fulfilled a median of 6.0 OCPD criteria compared with 3.0 among those with fair-to-good insight (*p* < 0.001; r = −0.39). Lochner et al. (2011) similarly found OCPD in OCD to be associated with greater symptom severity, psychiatric comorbidity, functional impairment, and poorer insight [48]. Coles et al. (2008) described a distinct clinical profile of co-occurring OCD and OCPD, including greater psychiatric comorbidity [58]. Fineberg et al. (2014) emphasized perfectionism, rigidity, and the need for control as central characteristics of OCPD, providing a relevant phenomenological context for our finding [59]. More specifically, Riddle et al. (2016) found the order/control dimension of OCPD to be associated with poor insight [60].

Among the five OCS dimensions, only hoarding was significantly more pronounced in the poor-to-absent insight group (median 1.0 vs. 0.0; *p* = 0.003; r = −0.33). This is consistent with previous observations [34,46] and, more specifically, with the analysis by Jakubovski et al. (2011), in which the association between hoarding and poor insight remained after adjustment for OCD severity, age, and sex [61]. Contamination/cleaning, taboo thoughts, doubts/checking, and symmetry/ordering did not distinguish the groups. Taboo thoughts were numerically less pronounced in patients with poor-to-absent insight, although this difference was not statistically significant (*p* = 0.058). Together with the heterogeneous findings of earlier studies [42,47], our results do not support a straightforward correspondence between obsessive–compulsive symptom content and insight.

Poor-to-absent insight was also accompanied by a higher psychiatric comorbidity count in the unadjusted between-group comparison, with a median count of 2.0 versus 1.0 (*p* = 0.035). This finding is consistent with earlier reports placing poor insight within a more clinically complex presentation of OCD [34,43,45,57]. Catapano et al. (2010) described greater psychiatric and personality-related burden among patients with poor insight [43], while Elvish et al. (2010) identified psychiatric comorbidity among the clinical characteristics associated with poor insight [57]. Notably, however, the higher psychiatric comorbidity count observed in our sample was not accompanied by greater current depressive symptom severity or higher YMRS scores. One possible explanation is that these measures capture different levels of clinical information. The psychiatric comorbidity count reflects broader diagnostic complexity, whereas HDRS and YMRS quantify current affective symptom severity at the time of assessment. Accordingly, the presence of psychiatric comorbidities need not be accompanied by greater current affective symptom intensity, particularly in a treatment-experienced clinical sample. This interpretation remains tentative, as the cross-sectional design does not allow treatment effects or longitudinal fluctuations in affective symptoms to be disentangled from these associations. The absence of a difference in depressive symptom severity contrasts with the meta-analytic association between greater depressive severity and poor insight reported by Gan et al. (2022) [15] and with the findings of de Avila et al. (2019) [34]. The latter study also reported a higher prevalence of bipolar disorder among patients with poor insight [34], while Marazziti et al. (2002) noted lower insight in patients with a history of recurrent manic or hypomanic episodes [40]. YMRS scores did not differentiate the insight groups in our sample; associations involving YMRS emerged in the subsequent dimensional and multivariable analyses and in the exploratory network analysis, particularly for BABS item 6. Although anger and aggressiveness are frequently described within the clinical phenomenology of OCD [17,18,19], alongside increased impulsivity [16], none of these dimensions differentiated patients with fair-to-good from those with poor-to-absent insight.

Among the sociodemographic characteristics, age was higher in the poor-to-absent insight group in the unadjusted between-group comparison (49.6 vs. 42.8 years; d = −0.55; *p* = 0.036), whereas sex, education, relationship status, and employment did not differ significantly between groups. This age difference is consistent with the observation by Guillén-Font et al. (2021) [23], whereas de Avila et al. (2019) and Ravi Kishore et al. (2004) did not report a significant association between age and insight [34,46]. Employment likewise did not distinguish the insight groups in our sample, despite previous reports linking poorer insight with unemployment [23,61]. These inconsistencies suggest that sociodemographic correlates of insight may depend on sample characteristics and analytical approach rather than representing uniform features across OCD populations. In the present analyses, the relevance of age was more apparent for BABS item 6, for which it retained a significant adjusted association, than for BABS total.

With regard to illness history and course, illness duration was numerically longer in the poor-to-absent insight group (26.0 vs. 21.0 years), although the difference did not reach statistical significance (*p* = 0.081). Treatment delay was comparable between groups, and a chronic course predominated in both, occurring in 91.7% of patients with fair-to-good insight and in all patients with poor-to-absent insight. This pattern only partly converges with the available literature. Guillén-Font et al. (2021) reported an association between poorer insight and a less favorable illness course [23], while Catapano et al. (2010) observed poorer longitudinal outcomes, including lower remission rates, among patients with poor insight [43].

### 5.2. Dimensional and Conditional Clinical Correlates of Insight

Extending the analysis beyond the categorical insight groups revealed both continuity and divergence across the full distribution of insight. After FDR correction, BABS total and BABS item 6 shared associations with age, illness duration, OCPD trait burden, and OCD severity; psychiatric comorbidity, hoarding, employment, and illness course were additionally associated with BABS total, and YMRS total with BABS item 6. For network modeling, Y-BOCS total was retained as the global severity measure in place of its structurally overlapping obsession and compulsion subscales. The subsequent conditional networks therefore provided a means of distinguishing associations directly connected with the insight outcomes from those embedded within the broader clinical configuration.

The strongest shared connection in both networks was between age and illness duration (partial r = 0.368). Longer illness duration was also linked to greater OCPD trait burden (partial r = 0.215 for BABS total and 0.219 for BABS item 6). Illness course formed part of the same configuration: episodic course was negatively connected with both illness duration and OCD severity, placing chronic course in the opposite direction, alongside longer-standing and more severe OCD. This organization is broadly compatible with longitudinal observations linking poor insight to a less favorable course [62]. It also accords with findings by Jakubovski et al. (2013), in which patients with particularly long-standing OCD maintained greater symptom severity over follow-up [14]. Interpretation of illness course in the present sample nevertheless requires caution, as only five participants had an episodic course.

A particularly coherent component of the network was organized around anankasticity. Greater OCPD trait burden was connected with longer illness duration, more pronounced hoarding, and higher levels of manic symptoms as indexed by YMRS scores. The OCPD–hoarding connections were closely comparable across the two networks (partial r = 0.188 and 0.205), as were those between OCPD traits and YMRS total (0.139 and 0.147). These relationships also showed high bootstrap stability, with the OCPD–illness duration, OCPD–hoarding, and OCPD–YMRS connections retained in more than 92% of resamples in both networks (Supplementary Table S2). Thus, the marked OCPD trait burden observed in the poor-to-absent insight group was not an isolated characteristic but formed part of a broader configuration involving chronicity, hoarding, and variation in manic symptom severity. Melca et al. (2015) provide a relevant clinical parallel, having reported greater hoarding severity and more frequent bipolar disorders among patients with co-occurring OCD and OCPD [63].

A second recurrent configuration involved OCD severity, psychiatric comorbidity, and occupational functioning. Greater OCD severity was positively connected with psychiatric comorbidity in both networks (partial r = 0.155 and 0.184), whereas employment was negatively connected with both OCD severity (−0.195 and −0.203) and psychiatric comorbidity (−0.169 and −0.177). These employment-related connections were among the more stable interrelations in both networks (Supplementary Table S2). This adds an important qualification to the categorical comparison: employment itself did not distinguish the insight groups and did not retain a direct connection with either insight outcome. Its network position was therefore more closely related to overall clinical burden and functioning than to insight per se.

Against this largely shared clinical background, the two insight outcomes occupied different conditional positions, as illustrated in Panel A of Figure 2 and Figure 3. For BABS total, OCD severity had by far the strongest direct connection with the outcome (partial r = 0.251), while all other direct connections were substantially weaker. For BABS item 6, the strongest direct connections instead involved YMRS total (partial r = 0.198), age (0.166), and OCD severity (0.140); OCPD traits and illness duration retained only weak direct connections, while psychiatric comorbidity, hoarding, employment, and illness course did not retain direct outcome connections. The principal direct associations also showed high bootstrap stability: the Y-BOCS total–BABS total edge was retained in 99.2% of resamples, the YMRS total–BABS item 6 edge in 95.2%, and the age–BABS item 6 edge in 93.3% (Supplementary Table S2).

Relative-importance analysis provided a complementary perspective by quantifying how model fit was distributed across seven clinically interpretable variables. For BABS total, the model explained 40.0% of the observed variance. OCD severity accounted for the largest share of the full-model R^2^ (37.2%), followed by manic symptoms (18.6%), OCPD traits (13.6%), and psychiatric comorbidity (11.6%); hoarding, illness duration, and age made smaller contributions. The predominance of OCD severity was also the most stable feature of the ranking: Y-BOCS total ranked first in 66.1% of bootstrap resamples and among the two leading contributors in 86.7% (Supplementary Table S3).

When the same seven variables were entered simultaneously, Y-BOCS total was the only predictor retaining a statistically significant adjusted association with BABS total (standardized β = 0.339; 95% CI 0.051–0.628; *p* = 0.021). OCPD traits, manic symptoms, age, illness duration, psychiatric comorbidity, and hoarding did not retain significant distinct associations. This does not conflict with their positions in the conditional network or their contributions to relative importance: network analysis describes conditional organization, relative-importance analysis partitions explained variance, and the fully adjusted model estimates distinct associations after simultaneous adjustment. Across these complementary approaches, OCD severity therefore emerged as the most consistent correlate of BABS total. Its association was also preserved in several prespecified sensitivity analyses, including adjustment for taboo thoughts and sex and analysis of BABS total after subtraction of item 6 (Supplementary Table S4). Although Y-BOCS and BABS target different constructs, both are clinician-rated measures of related aspects of current OCD psychopathology obtained within the same assessment context; shared-method variance and partial conceptual overlap may therefore have contributed to their association. The present data do not permit the magnitude of this measurement-related contribution to be quantified.

A more differentiated pattern emerged for BABS item 6, with the relative prominence of clinical correlates varying across cumulative thresholds. Age contributed most at the lowest threshold (40.3%), OCPD trait count at the intermediate threshold (28.9%), and YMRS total at >2 versus 0–2 (53.8%). Bootstrap rankings showed a similar pattern across thresholds, with the same variables most frequently ranking first at the corresponding thresholds (Supplementary Table S3). Given the small numbers at the upper thresholds, however, the apparent differences across thresholds should be regarded as exploratory and may partly reflect sampling variability rather than a stable threshold-dependent hierarchy. The highest primary relative-importance contrast included only 11 participants above the threshold, limiting the precision of this finding.

The fully adjusted ordinal model did not reduce this pattern to OCD severity. Older age remained associated with higher BABS item 6 categories (*p* = 0.020), whereas YMRS total became significant only at the upper cumulative thresholds (both *p* < 0.01). These threshold-specific associations should be interpreted cautiously, particularly at the highest threshold, which was based on only four participants. Nevertheless, the broader pattern was supported by several alternative model specifications (Supplementary Table S4). Given the generally low YMRS scores in the sample and the absence of a between-group difference, this finding is better understood as reflecting variation in manic symptom severity within a predominantly low-symptom range rather than clinically manifest mania.

Taken together, these analyses indicate a broadly shared pattern of clinical associations within which the two insight outcomes nevertheless occupy different positions. OCD severity remained the central and most consistent correlate of BABS total, whereas age and higher levels of manic symptoms, as indexed by YMRS scores, became more prominent at higher levels of BABS item 6. The implications of this partial convergence and divergence for the conceptualization and clinical assessment of insight are considered in the following section.

### 5.3. Conceptual and Clinical Implications

Existing conceptualizations approach insight at different levels, reflecting the difficulty of treating it as a unitary psychopathological property. David (1990) described insight as a multicomponent construct encompassing recognition of illness, attribution of symptoms to that illness, and acknowledgment of the need for treatment [29]. Marková et al. (2009), addressing insight specifically in OCD, argued that it is better understood as a form of self-knowledge and a mental state than as a single symptom or unitary dimension of psychopathology [33]. De Avila et al. (2019) further situated poor insight within a broader process of self-appraisal involving evaluation of one’s own experiences, anticipation of their consequences, and perception of one’s capacity to act [34].

The partial divergence between BABS total and BABS item 6 is particularly informative in this context. Their different positions within an otherwise broadly shared clinical architecture, understood here descriptively as a pattern of clinical interrelationships rather than a mechanistic structure, do not imply two distinct forms of insight. Because BABS item 6 contributes to the BABS total score, the two outcomes are related by construction, and differences in their association patterns should not be interpreted as evidence of independent constructs. Some of the observed divergence may also reflect differences in scale range, ordinal structure, and measurement precision rather than distinct underlying constructs. At the same time, BABS total integrates several components of insight, whereas item 6 captures a single component; their clinical correlates may therefore differ without implying distinct underlying phenomena. Shimshoni et al. (2011) found substantial agreement among several insight measures in OCD despite partly measure-specific relationships with clinical and demographic characteristics [64]. Studies using the BABS itself provide a similar perspective. Eisen et al. (2004) assessed both global insight and individual components of insight [65], while Phillips et al. (2012), in 211 patients with OCD, showed that although OCD severity was related to insight, severity and insight/delusionality were not equivalent constructs [66].

The phenomenology of obsessive–compulsive beliefs provides another perspective. Kozak and Foa (1994) challenged a simple dichotomy between preserved and absent insight and instead proposed a continuum of belief strength extending from typical obsessional beliefs through overvalued ideas toward beliefs approaching delusional certainty [67]. Brakoulias and Starcevic (2011) broadened this characterization by emphasizing that OCD-related beliefs may vary simultaneously in conviction, fixity, fluctuation, resistance, awareness of their inaccuracy, and attribution to illness [68]. These accounts are compatible with the present findings, which suggest that clinical variation in insight is not adequately captured by a single gradient of symptom severity or belief conviction. Rather than challenging a continuum model, our findings suggest that comparable levels of insight may occur within partly different clinical contexts. The lack of a straightforward relationship between most OCS dimensions and categorical insight further suggests that symptom content itself may be less informative than the manner in which patients relate to their obsessive–compulsive beliefs and experiences: the strength with which they endorse them, their openness to alternative interpretations, and their capacity to attribute these experiences to the disorder.

Insight also appears to vary within individuals rather than functioning solely as a stable characteristic. Experience-sampling studies by Landmann et al. (2019) and Bischof et al. (2025) demonstrated substantial within-person variation in momentary aspects of insight over periods of several days [69,70]. Over a substantially longer interval, Wolf et al. (2023) observed changes in insight in 70% of participants with available longitudinal data, particularly among those entering follow-up with poor insight; changes in insight accompanied changes in OCD severity, although earlier changes in insight did not clearly predict subsequent severity [71]. Earlier observations of improvement in insight during treatment point in the same direction [42]. Together, these studies support a view of insight as both multifaceted and dynamic, with its clinical expression capable of changing alongside the broader clinical state.

The cross-sectional design of the present study cannot determine whether the pattern of clinical associations identified here changes over time. The longitudinal variability described above nevertheless provides an empirical basis for considering the present findings from a temporal perspective. Changes in insight may involve not only variation in its overall level, but also shifts in the relative prominence of associated clinical characteristics and in the configuration within which different aspects of insight are expressed. Prospective studies combining repeated assessment of insight with parallel measurement of the principal elements identified here could establish which relationships remain relatively stable, which covary with clinical state, and whether changes in their configuration precede changes in insight, accompany them, or follow them.

The mechanisms underlying this heterogeneity remain beyond the scope of the present data. At the cognitive level, the inference-based account of OCD emphasizes how imagined possibilities may acquire greater weight than directly available information in obsessional doubt [72], while neuropsychological findings have implicated executive and broader cognitive processes in poorer insight [36]. Broekhuizen et al. (2023) further identified differences in brain activation during symptom provocation involving emotional and sensory processing and cognitive control that were not explained by OCD severity alone [73]. These lines of evidence are consistent with the possibility that the heterogeneity of insight reflects multiple underlying processes rather than a single explanatory mechanism.

From a clinical perspective, the convergence of findings across complementary analytic approaches suggests that poorer insight should be considered within the broader psychopathological and clinical context of OCD rather than in isolation. Previous studies have similarly linked poorer insight to greater symptom severity, clinical complexity, impaired functioning, and a less favorable illness course [23,34,43,46,57,61]. Such characteristics may warrant greater clinical attention to insight, but should not be regarded as proxies for insight itself, particularly because OCD severity and insight, although related, are not equivalent constructs [66]. Clinical evaluation should therefore consider both the overall level of insight and the possibility that its individual aspects may be unevenly expressed.

The potential clinical relevance of insight may also extend to treatment engagement and the interpretation of apparent treatment resistance. Estimates of treatment-resistant OCD vary substantially, and inadequate treatment exposure or poor adherence may contribute to pseudo-resistance [74]. In psychotherapy, a meta-analysis of 123 studies found refusal and dropout rates of approximately 16%, while better adherence to between-session therapeutic tasks was associated with greater symptom improvement [75]. Some OCD studies have associated poor insight with less favorable treatment outcomes [43,46], whereas others have not confirmed this relationship [42,49]. Although adherence and treatment response were not assessed in the present study, these observations raise the possibility that insight may be clinically relevant when treatment engagement is limited or apparent treatment resistance is being considered. These potential relationships require prospective investigation.

More broadly, future research on insight in OCD may benefit from moving beyond static associations between individual clinical characteristics and insight measured at a single time point. Longitudinal approaches tracking insight alongside changes in psychopathology, symptom phenomenology, and treatment course could clarify which associations represent relatively stable characteristics and which vary with the clinical state. Such work may ultimately help determine whether insight is best understood not only as a level of awareness or conviction, but also as a dynamic clinical phenomenon whose expression changes within the broader course of OCD.

## 6. Conclusions

1. Poor-to-absent insight was present in one quarter of this clinical sample.
2. OCD severity was the most consistent correlate of poorer global insight and the only independently significant correlate of BABS total in the fully adjusted model, whereas anankastic traits and other clinical characteristics were represented in broader dimensional and network associations.
3. Categorical and dimensional analyses provided complementary perspectives, and BABS total and BABS item 6 showed partly different association profiles, although item 6 forms part of the total score.
4. These findings support direct assessment of insight alongside consideration of the broader psychopathological and clinical context.

## 7. Limitations

1. The study was conducted in a single clinical centre and included a relatively modest sample of 80 treatment-experienced patients, including 20 with poor-to-absent insight. The predominance of chronic and relatively severe OCD may limit the precision of some estimates and the generalisability of the findings to milder, untreated, community-based, or earlier-stage populations. Recruitment from both inpatient and outpatient settings may limit generalizability across clinical settings.
2. The cross-sectional design precludes conclusions regarding temporal direction or causality. The study cannot determine whether the clinical characteristics associated with poorer insight preceded it, developed alongside it, or reflected shared underlying processes. Accordingly, the pattern of associations identified here should be regarded as descriptive and hypothesis-generating rather than as evidence of an underlying causal structure.
3. Some clinically relevant categories were sparsely represented. Only five participants had an episodic course, 11 had BABS item 6 scores >2, and only four scored >3, reducing the precision of estimates involving these variables. YMRS scores were also generally low, so associations with manic symptoms should not be extrapolated to clinically manifest mania.
4. Insight was assessed with a single clinician-administered instrument. BABS item 6 is a single ordinal item and contributes to the BABS total score; the two outcomes are therefore related by construction and should not be regarded as independent replications. In addition, the five checklist-derived OCD dimensions represent aggregated symptom domains and may not capture associations involving more specific symptom content.
5. Between-group comparisons were not adjusted for multiple testing, whereas multiplicity was controlled using false-discovery-rate correction in the whole-sample dimensional screening. Secondary categorical findings should therefore be interpreted in conjunction with their effect sizes and with the extent to which they converged with subsequent dimensional analyses.
6. The multistage analytical strategy was applied within the same sample. Bootstrap procedures provided information on the internal stability of the network and relative-importance findings but do not establish external reproducibility. Independent replication will therefore be required to assess their generalisability.
7. The psychiatric comorbidity measure represented a count across four diagnostic categories and did not capture the full range, severity, or differential clinical significance of individual comorbid disorders. Additional developmental, cognitive, metacognitive, treatment-related, or neurobiological factors not assessed in the present study may also contribute to insight.
8. Current pharmacotherapy was not included in the analyses and should therefore be considered when interpreting associations involving symptom severity.

## Figures and Tables

**Table 1:** Sociodemographic and clinical characteristics of the study sample (N = 80).

Characteristic	n (%)	Mean ± SD or Median (Q1–Q3)	Range
**Panel A. Sociodemographic and clinical characteristics**			
Sociodemographic characteristics			
Age, years		44.5 ± 12.6	18–73
Sex			
Female	44 (55.0)		
Male	36 (45.0)		
Education			
Primary	1 (1.3)		
Vocational	9 (11.3)		
Secondary	24 (30.0)		
Tertiary	46 (57.5)		
Relationship status			
In a relationship	51 (63.8)		
Not in a relationship	29 (36.3)		
Employment			
Active	49 (61.3)		
Inactive	31 (38.8)		
Clinical course and time-related variables			
Illness duration, years		22.2 ± 11.1	1–55
Treatment delay, years		10.0 (5.0–19.5)	0–44
Treatment duration, years		7.0 (1.5–15.0)	0–35
Course of OCD			
Chronic, no remission lasting ≥12 months	75 (93.8)		
Episodic, ≥1 remission lasting ≥12 months	5 (6.3)		
OCD severity			
Y-BOCS total		22.7 ± 7.1	8–38
Y-BOCS obsessions		12.0 (9.0–15.0)	4–19
Y-BOCS compulsions		10.9 ± 3.8	3–19
OCD severity category			
Mild	15 (18.8)		
Moderate	25 (31.3)		
Severe	32 (40.0)		
Extreme	8 (10.0)		
Psychiatric comorbidity			
Psychiatric comorbidity count		2.0 (1.0–2.0)	0–3
Number of psychiatric comorbidities			
0	10 (12.5)		
1	28 (35.0)		
2	35 (43.8)		
3	7 (8.8)		
**Panel B. Checklist-derived OCD dimensions**	**%**	**Median (Q1–Q3)**	**Range**
Contamination/cleaning	59.4	1.0 (0.0–2.0)	0–2
Taboo thoughts	52.1	2.0 (1.0–2.0)	0–3
Doubts/checking	66.3	1.0 (1.0–2.0)	0–2
Symmetry/ordering	58.4	2.0 (2.0–3.0)	0–4
Hoarding	36.3	0.0 (0.0–1.0)	0–2

Note. Values are presented as n (%), mean ± SD, or median (Q1–Q3), as appropriate. Abbreviations: OCD, obsessive–compulsive disorder; OCPD, obsessive–compulsive personality disorder; Q1, lower quartile; Q3, upper quartile; SD, standard deviation; Y-BOCS, Yale–Brown Obsessive–Compulsive Scale.

**Table 2:** Descriptive statistics for psychometric measures in the study sample (N = 80).

Measure	Total Sample	Min–Max	Shapiro–Wilk *p*
Insight			
BABS total	10.5 (6.0–12.5)	1.0–21.0	0.037
BABS item 6	2.0 (1.0–2.0)	0.0–4.0	<0.001
Affective symptoms			
HDRS total	8.0 (5.0–13.5)	0.0–36.0	<0.001
YMRS total	2.0 (0.0–5.0)	0.0–24.0	<0.001
Personality traits			
OCPD traits	4.0 (2.0–5.0)	0.0–8.0	0.006
Impulsivity			
BIS total	64.0 ± 11.3	41.0–96.0	0.052
BIS-Cog	18.0 (16.0–20.0)	10.0–28.0	0.043
BIS-Mot	20.0 (18.0–23.0)	13.0–36.0	<0.001
BIS-Plan	24.0 (21.0–28.0)	12.0–49.0	0.004
Aggression			
BPAQ total	78.5 ± 18.3	41.0–126.0	0.740
BPAQ-PA	17.0 (13.0–22.0)	9.0–37.0	<0.001
BPAQ-VA	14.5 (11.0–17.0)	7.0–24.0	0.045
BPAQ-A	21.7 ± 5.8	1.0–35.0	0.130
BPAQ-H	23.7 ± 6.9	8.0–37.0	0.085

Note. Values are presented as mean ± SD or median (Q1–Q3), as appropriate. Min–Max indicates the observed range. *p* refers to the Shapiro–Wilk test of normality; *p* < 0.05 indicates a statistically significant departure from normality. Abbreviations: BABS, Brown Assessment of Beliefs Scale; BIS, Barratt Impulsiveness Scale; BIS-Cog, cognitive/attentional impulsivity; BIS-Mot, motor impulsivity; BIS-Plan, non-planning impulsivity; BPAQ, Buss–Perry Aggression Questionnaire; BPAQ-A, anger; BPAQ-H, hostility; BPAQ-PA, physical aggression; BPAQ-VA, verbal aggression; HDRS, Hamilton Depression Rating Scale; OCPD traits, number of obsessive–compulsive personality disorder traits; SD, standard deviation; YMRS, Young Mania Rating Scale.

**Table 3:** Sociodemographic and clinical characteristics by insight group (N = 80).

Variable	Fair-to-Good Insight (N = 60)	Poor-to-Absent Insight (N = 20)	*p*-Value	Effect Size
Insight				
BABS total	8.5 (4.0–11.0)	16.0 (15.0–17.5)	<0.001	−0.75 (r)
BABS item 6	1.0 (0.0–2.0)	2.5 (2.0–3.0)	<0.001	−0.63 (r)
Sociodemographics				
Age, years	42.8 ± 11.8	49.6 ± 13.8	0.036	−0.55 (d)
Sex, n (%)			0.604	0.06 (V)
Female	34 (56.7)	10 (50.0)		
Male	26 (43.3)	10 (50.0)		
Education, n (%)			0.680	0.14 (V)
Primary	1 (1.7)	0 (0.0)		
Vocational	8 (13.3)	1 (5.0)		
Secondary	17 (28.3)	7 (35.0)		
Tertiary	34 (56.7)	12 (60.0)		
Relationship status, n (%)			0.140	0.17 (V)
In a relationship	41 (68.3)	10 (50.0)		
Not in a relationship	19 (31.7)	10 (50.0)		
Employment, n (%)			0.233	0.13 (V)
Active	39 (65.0)	10 (50.0)		
Inactive	21 (35.0)	10 (50.0)		
OCD severity				
Y-BOCS total	21.0 ± 6.6	27.7 ± 6.4	<0.001	−1.02 (d)
Y-BOCS obsessions	12.0 (8.0–13.0)	14.5 (12.5–16.0)	<0.001	−0.40 (r)
Y-BOCS compulsions	10.1 ± 3.5	13.5 ± 3.6	<0.001	−0.97 (d)
Clinical course and time-related variables				
Illness duration, years	21.0 ± 10.0	26.0 ± 13.6	0.081	−0.46 (d)
Treatment delay, years	10.0 (5.0–18.0)	8.5 (7.0–20.0)	0.889	0.02 (r)
Treatment duration, years	7.0 (1.5–13.5)	10.0 (1.5–20.0)	0.370	−0.10 (r)
Course of OCD, n (%)			0.324	0.15 (V)
Chronic	55 (91.7)	20 (100.0)		
Episodic	5 (8.3)	0 (0.0)		
Comorbidity and personality				
Psychiatric comorbidity count	1.0 (1.0–2.0)	2.0 (1.5–2.0)	0.035	−0.24 (r)
OCPD trait count	3.0 (2.0–4.0)	6.0 (4.0–7.5)	<0.001	−0.39 (r)
Affective symptoms				
HDRS total	8.5 (5.0–12.0)	7.0 (4.5–15.0)	0.824	−0.03 (r)
YMRS total	2.0 (0.0–5.0)	2.5 (1.0–9.5)	0.167	−0.16 (r)
Impulsivity				
BIS total	63.0 ± 11.0	66.8 ± 12.0	0.197	−0.34 (d)
BIS-Cog	17.5 (16.0–20.0)	18.0 (15.0–20.5)	0.951	−0.01 (r)
BIS-Mot	20.0 (18.0–22.5)	20.0 (19.0–25.0)	0.257	−0.13 (r)
BIS-Plan	23.5 (20.0–28.0)	24.0 (22.5–30.0)	0.330	−0.11 (r)
Aggression				
BPAQ total	78.4 ± 18.6	78.6 ± 18.0	0.978	−0.01 (d)
BPAQ-PA	17.0 (13.0–22.0)	16.5 (13.0–24.0)	0.898	0.02 (r)
BPAQ-VA	14.0 (11.0–17.0)	15.5 (12.5–18.0)	0.210	−0.14 (r)
BPAQ-A	22.0 ± 5.9	21.2 ± 5.8	0.576	0.14 (d)
BPAQ-H	24.0 ± 7.0	22.9 ± 6.7	0.546	0.16 (d)
Checklist-derived OCD dimensions				
Contamination/cleaning	2.0 (0.0–2.0)	1.0 (0.0–2.0)	0.211	0.14 (r)
Taboo thoughts	2.0 (1.0–2.0)	1.0 (0.5–2.0)	0.058	0.21 (r)
Doubts/checking	1.0 (1.0–2.0)	1.0 (1.0–2.0)	0.956	0.01 (r)
Symmetry/ordering	2.0 (2.0–3.0)	2.5 (2.0–3.5)	0.669	−0.05 (r)
Hoarding	0.0 (0.0–1.0)	1.0 (0.5–2.0)	0.003	−0.33 (r)

Note. Fair-to-good insight was defined as BABS total ≤12; poor-to-absent insight was defined as BABS total >12. Values are presented as mean ± SD, median (Q1–Q3), or n (%), as appropriate. Effect sizes are reported as r for Mann–Whitney U tests, Cohen’s d for Student’s t tests, and Cramér’s V for categorical variables. Negative r and d values indicate higher values in the poor-to-absent insight group because the fair-to-good insight group was entered first. Abbreviations: BABS, Brown Assessment of Beliefs Scale; BIS, Barratt Impulsiveness Scale; BIS-Cog, cognitive/attentional impulsivity; BIS-Mot, motor impulsivity; BIS-Plan, non-planning impulsivity; BPAQ, Buss–Perry Aggression Questionnaire; BPAQ-A, anger; BPAQ-H, hostility; BPAQ-PA, physical aggression; BPAQ-VA, verbal aggression; HDRS, Hamilton Depression Rating Scale; OCD, obsessive–compulsive disorder; OCPD, obsessive–compulsive personality disorder; SD, standard deviation; Y-BOCS, Yale–Brown Obsessive–Compulsive Scale; YMRS, Young Mania Rating Scale.

**Table 4:** Complementary multivariable models of BABS total and BABS item 6 (N = 80).

**Panel A. BABS Total: Multiple Linear Regression**			
Variable	Standardized β	95% CI	***p***
Y-BOCS total	0.339	0.051–0.628	0.021
OCPD trait count	0.100	−0.161–0.362	0.453
YMRS total	0.222	−0.071–0.515	0.138
Age	0.081	−0.200–0.362	0.572
Illness duration	0.052	−0.203–0.307	0.689
Psychiatric comorbidity count	0.100	−0.242–0.441	0.566
Hoarding	0.065	−0.115–0.245	0.480
Model fit: R^2^ = 0.400; adjusted R^2^ = 0.342; global *p* < 0.001.			
**Panel B. BABS Item 6: Partial Proportional-Odds Ordinal Logistic Regression**			
Variable or cumulative contrast	OR per 1-SD increase	95% CI	***p***
Y-BOCS total	1.588	0.920–2.741	0.097
OCPD trait count	1.370	0.792–2.369	0.260
Age	1.975	1.115–3.499	0.020
Illness duration	1.127	0.643–1.975	0.676
Psychiatric comorbidity count	0.859	0.498–1.481	0.585
Hoarding	0.885	0.547–1.430	0.617
YMRS total: threshold-specific cumulative effects			
BABS item 6 >0 vs. 0	1.329	0.572–3.088	0.509
BABS item 6 >1 vs. 0–1	1.619	0.791–3.316	0.187
BABS item 6 >2 vs. 0–2	3.021	1.452–6.286	0.003
BABS item 6 >3 vs. 0–3	2.622	1.285–5.350	0.008
Model fit: LR χ^2^(10) = 33.968; global *p* < 0.001; McFadden pseudo-R^2^ = 0.151.			

Note. The same seven clinical variables were entered simultaneously in both models. In Panel (**A**), estimates are standardized β coefficients; 95% confidence intervals and *p* values are based on HC3 heteroskedasticity-robust standard errors. In Panel (**B**), ORs are reported per 1-SD increase. Threshold-specific ORs are shown for YMRS total because the proportional-odds assumption was violated for this variable. Abbreviations: BABS, Brown Assessment of Beliefs Scale; CI, confidence interval; LR, likelihood ratio; OCPD, obsessive–compulsive personality disorder; OR, odds ratio; SD, standard deviation; Y-BOCS, Yale–Brown Obsessive–Compulsive Scale; YMRS, Young Mania Rating Scale.

## Data Availability

The data presented in this study are available on reasonable request from the corresponding author. The data are not publicly available due to participant privacy and restrictions arising from the scope of the informed consent obtained.

## Supplementary Materials

The following supporting information can be downloaded at: https://www.mdpi.com/article/10.3390/jcm15187289/s1, Supplementary Material S1: Supplementary Methods and Results, including dimensional screening, conditional-network analysis, relative-importance analysis, bootstrap stability, sensitivity analyses, Tables S1–S4, and Figure S1; Supplementary Material S2: Diagnostic Verification Form (English version); Supplementary Table S1. Outcome-specific Spearman associations of all 30 clinical candidates with BABS total and BABS item 6 (N = 80); Supplementary Table S2. Conditional-network edge estimates and bootstrap stability (N = 80); Supplementary Table S3. Bootstrap stability of relative-importance estimates and rankings (N = 80); Supplementary Table S4. Conventional multivariable and planned sensitivity analyses; Supplementary Figure S1. Full Spearman association matrix of insight measures and clinical variables (N = 80); Supplementary Data S1: Complete 32 × 32 matrices of Spearman r, unadjusted p, and Benjamini–Hochberg-adjusted q values, together with outcome-specific correlation summaries and coding notes; Supplementary Data S2: Complete numerical outputs for conditional-network, relative-importance, complementary multivariable, diagnostic, bootstrap, and sensitivity analyses.
