# Supplementary material for: Clinical Correlates of Poor Insight in Obsessive–Compulsive Disorder: An Integrative Cross-Sectional Analysis
Source: J Clin Med. 2026 Sep 19;15(18):7289. doi: 10.3390/jcm15187289 (PMC13607875; doi:10.3390/jcm15187289)
Supplement: Supplementary file 1 [file jcm-15-07289-s001.zip › Supplementary Material S1.pdf]

**Supplementary Material S1**

# Clinical Correlates of Poor Insight in Obsessive–Compulsive Disorder: An Integrative Cross-Sectional Analysis

The Supplementary Methods provide detailed specifications of the dimensional screening, conditional-network analysis, relative-importance decomposition, bootstrap procedures, and complementary sensitivity analyses summarized in Section 3.2 of the main manuscript.

**Contents**

Supplementary Methods
Supplementary Results
Supplementary Table S1. Outcome-specific Spearman associations of all 30 clinical candidates
Supplementary Table S2. Conditional-network edge estimates and bootstrap stability
Supplementary Table S3. Bootstrap stability of relative-importance estimates and rankings
Supplementary Table S4. Conventional multivariable and planned sensitivity analyses
Supplementary Figure S1. Full Spearman association matrix
Supplementary Data S1 and S2. Complete numerical outputs supplied as Excel files

## Supplementary Methods

### Candidate-variable screening and full correlation matrix

Following the categorical comparison of fair-to-good and poor-to-absent insight in the main manuscript, dimensional screening was performed in the full sample (N = 80). Spearman rank correlations were calculated between each insight-related outcome (BABS total and BABS item 6) and 30 sociodemographic, clinical, affective, personality, impulsivity, aggression, and checklist-derived symptom variables. Benjamini–Hochberg correction was applied separately across the 30 candidate associations for each outcome, with the false discovery rate controlled at 5%. Outcome-specific FDR-adjusted q values are reported in Supplementary Table S1 and Supplementary Data S1.

A separate exploratory 32 × 32 matrix was generated for all study variables, including both insight outcomes. For Supplementary Figure S1, Benjamini–Hochberg correction was applied across all 496 unique pairwise correlations. Complete Spearman r, unadjusted p, and both outcome-specific and full-matrix q values are supplied in Supplementary Data S1. The two multiplicity families were therefore distinct:

Supplementary Table S1 uses outcome-specific correction across 30 candidates, whereas Supplementary Figure S1 uses correction across all 496 unique pairwise correlations.

**Conditional-network analysis**

The network stage used the union of variables whose association with BABS total or BABS item 6 remained significant after outcome-specific FDR correction. Because Y-BOCS total, obsession, and compulsion scores are structurally overlapping measures, only Y-BOCS total was retained for the network representation. The common candidate set comprised age, employment status, illness duration, episodic versus chronic course, Y-BOCS total, psychiatric comorbidity count, OCPD trait count, hoarding, and YMRS total. The same candidate set was used for both outcome-centred networks.

Variables were transformed using a rank-based Gaussian transformation and entered into a graphical-lasso model. The regularization penalty was selected by five-fold cross-validation (alpha = 0.124 for BABS total and alpha = 0.106 for BABS item 6). Network edges represent partial correlations between two variables after accounting for all remaining nodes in the same network. Edge stability was assessed using 1,000 nonparametric bootstrap resamples with the full-sample penalty retained. For each edge, selection frequency, sign consistency, the bootstrap median partial correlation, and the 2.5th and 97.5th percentiles were recorded. Exact estimates are reported in Supplementary Table S2 and Supplementary Data S2.

**Relative-importance analysis**

The final integrative stage quantified the relative contribution of seven clinically interpretable and nonredundant variables: Y-BOCS total, OCPD trait count, YMRS total, age, illness duration, psychiatric comorbidity count, and hoarding. Employment and episodic course remained part of the broader network architecture and were examined in extended sensitivity models rather than in the primary relative-importance analysis; only five participants had an episodic course. No stepwise selection procedure was used.

For BABS total, relative importance was estimated by LMG/Shapley decomposition of $R^2$ from the seven-predictor ordinary least-squares model. Each LMG/Shapley value represents the average incremental contribution of a predictor to $R^2$ across all possible predictor subsets and entry orders. Contributions sum to the full-model $R^2$ and are expressed as percentages of that $R^2$.

Because the proportional-odds assumption was violated for YMRS total in the ordinal analysis of BABS item 6, relative importance was evaluated using cumulative binary contrasts rather than by decomposing a partial proportional-odds model. Logistic models were fitted for BABS item 6 >0 versus 0, >1 versus 0–1, and >2 versus 0–2; the corresponding numbers above threshold were 64, 42, and 11. McFadden pseudo-$R^2$ was decomposed using the same LMG/Shapley principle. The >3 versus 0–3 contrast was not included in the primary relative-importance analysis because only four participants exceeded this threshold. Relative-

importance percentages are interpreted within each model and are not directly comparable as absolute explained variance between continuous and binary outcomes.

### Bootstrap stability and complementary analyses

Relative-importance stability was assessed using 1,000 nonparametric bootstrap resamples with a fixed random seed (20260811). All subset models and relative-importance values were recomputed within each resample. For each predictor, the bootstrap median, percentile 95% interval, frequency of ranking first, and frequency of ranking among the two leading contributors were recorded. Rank frequencies were calculated among estimable resamples.

Conventional fully adjusted models were retained as complementary analyses. BABS total was analysed using ordinary least-squares regression with HC3 heteroskedasticity-robust standard errors, and BABS item 6 using a partial proportional-odds model that allowed the YMRS coefficient to vary across cumulative thresholds. Continuous predictors were standardized before conventional multivariable modeling. Additional prespecified sensitivity analyses comprised replacement of Y-BOCS total by its obsession and compulsion subscales; addition of employment and episodic course; addition of taboo-thought severity; adjustment for sex; analysis of BABS total after subtraction of item 6; alternative categorization of BABS item 6; and ridge and elastic-net coefficient-stability checks. Complete diagnostics and numerical outputs are supplied in Supplementary Data S2.

All statistical tests were two-sided. Statistical significance was set at $p < 0.05$. For the outcome-specific dimensional screening analyses, FDR-adjusted q values $< 0.05$ were considered statistically significant.

## Supplementary Results

### Dimensional screening

After outcome-specific FDR correction, 10 variables remained associated with BABS total and 7 remained associated with BABS item 6. For BABS total these were age, employment status, illness duration, episodic course, Y-BOCS total, Y-BOCS obsessions, Y-BOCS compulsions, psychiatric comorbidity count, OCPD trait count, and hoarding. For BABS item 6 they were age, illness duration, Y-BOCS total, Y-BOCS obsessions, Y-BOCS compulsions, OCPD trait count, and YMRS total. Complete screening results are reported in Supplementary Table S1.

### Conditional clinical architecture

In the BABS total network, the strongest direct conditional connection was observed for Y-BOCS total (partial $r = 0.251$; selected in 99.2% of bootstrap samples). Smaller direct connections were observed for

OCPD traits (partial r = 0.117; 87.2%), YMRS total (0.098; 75.7%), psychiatric comorbidity count (0.093; 74.3%), episodic course (−0.095; 78.9%), hoarding (0.058; 70.8%), age (0.033; 52.0%), and illness duration (0.015; 51.8%); employment did not retain a direct outcome connection.

The strongest conditional interrelation between clinical variables was age with illness duration (partial r = 0.368), followed by illness duration with OCPD traits (0.215), employment with Y-BOCS total (−0.195), age with psychiatric comorbidity count (0.188), OCPD traits with hoarding (0.188), employment with psychiatric comorbidity count (−0.169), Y-BOCS total with psychiatric comorbidity count (0.155), and OCPD traits with YMRS total (0.139).

In the BABS item 6 network, the strongest direct conditional connections were YMRS total (partial r = 0.198; selected in 95.2% of bootstrap samples), age (0.166; 93.3%), and Y-BOCS total (0.140; 88.7%). Direct connections with OCPD traits (0.046; 62.9%) and illness duration (0.010; 54.3%) were weak; psychiatric comorbidity count, hoarding, employment, and episodic course did not retain direct outcome connections.

The conditional interrelations between the clinical variables substantially overlapped with the BABS total network. The strongest was again age with illness duration (partial r = 0.368), followed by illness duration with OCPD traits (0.219), OCPD traits with hoarding (0.205), employment with Y-BOCS total (−0.203), age with psychiatric comorbidity count (0.191), Y-BOCS total with psychiatric comorbidity count (0.184), employment with psychiatric comorbidity count (−0.177), and OCPD traits with YMRS total (0.147).

**Relative importance within the integrated clinical configuration**

For BABS total, the seven-variable model explained 40.0% of the observed variance ($R^2 = 0.400$). Y-BOCS total had the largest relative contribution (37.2% of the full-model $R^2$), followed by YMRS total (18.6%), OCPD trait count (13.6%), psychiatric comorbidity count (11.6%), hoarding (7.1%), illness duration (6.3%), and age (5.6%).

The relative-importance distribution differed across cumulative BABS item 6 contrasts. For >0 versus 0, age had the largest contribution (40.3%), followed by illness duration (25.6%). For >1 versus 0–1, OCPD trait count (28.9%) and Y-BOCS total (24.7%) had the largest contributions. For >2 versus 0–2, YMRS total had the largest contribution (53.8%), followed by age (20.0%) and Y-BOCS total (11.2%).

Bootstrap analyses supported the principal ranking patterns. Y-BOCS total ranked first for BABS total in 66.1% of resamples and among the two leading contributors in 86.7%. Age ranked first for BABS item 6 >0 in 51.1% and among the top two in 74.7%; OCPD trait count did so for >1 in 41.2% and 64.7%, respectively. For >2, YMRS total ranked first in 70.9% and among the top two in 85.5%. All 1,000 bootstrap resamples were estimable for each of the four primary relative-importance models.

**Conventional multivariable and sensitivity analyses**

The conventional seven-variable BABS total model explained 40.0% of the observed variance (adjusted $R^2 = 0.342$; global $p < 0.001$). Y-BOCS total retained a statistically significant adjusted association (standardized $\beta = 0.339$, 95% CI 0.051–0.628, $p = 0.021$); the remaining individual predictors were not significant.

In the partial proportional-odds model for BABS item 6, age showed a positive proportional association (OR = 1.975, 95% CI 1.115–3.499, $p = 0.020$). The YMRS effect varied across thresholds and was significant for >2 versus 0–2 (OR = 3.021, 95% CI 1.452–6.286, $p = 0.003$) and >3 versus 0–3 (OR = 2.622, 95% CI 1.285–5.350, $p = 0.008$).

Prespecified sensitivity analyses were broadly compatible with the principal pattern. Y-BOCS total remained associated with BABS total after adjustment for taboo-thought severity ($p = 0.026$), after adjustment for sex ($p = 0.025$), and after subtraction of BABS item 6 from the BABS total score ($p = 0.014$). For BABS item 6, YMRS total remained significant in several alternative specifications, including the extended model ($p = 0.009$), the model including taboo-thought severity ($p = 0.013$), the sex-adjusted model ($p = 0.007$), and the four-category partial proportional-odds model for the >2 contrast ($p = 0.003$). Age also remained significant in several alternative BABS item 6 specifications. Complete estimates are reported in Supplementary Table S4 and Supplementary Data S2.

Supplementary Table S1. Outcome-specific Spearman associations of all 30 clinical candidates with BABS total and BABS item 6 (N = 80).

| Variable | BABS total r | p | FDR q | BABS item 6 r | p | FDR q |
|---|---|---|---|---|---|---|
| Age | 0.321 | 0.004 | 0.014 | 0.369 | <0.001 | 0.010 |
| Sex (male=1) | 0.023 | 0.840 | 0.969 | 0.096 | 0.396 | 0.658 |
| Education | 0.130 | 0.249 | 0.373 | 0.171 | 0.130 | 0.300 |
| Relationship (yes=1) | -0.192 | 0.088 | 0.164 | -0.062 | 0.585 | 0.746 |
| Employment (active=1) | -0.286 | 0.010 | 0.030 | -0.145 | 0.198 | 0.396 |
| Illness duration | 0.336 | 0.002 | 0.010 | 0.302 | 0.006 | 0.032 |
| Treatment delay | 0.123 | 0.278 | 0.397 | 0.162 | 0.150 | 0.322 |
| Treatment duration | 0.153 | 0.176 | 0.310 | 0.092 | 0.416 | 0.658 |
| Episodic course | -0.302 | 0.006 | 0.022 | -0.197 | 0.080 | 0.219 |
| Y-BOCS total | 0.533 | <0.001 | <0.001 | 0.361 | 0.001 | 0.010 |
| Y-BOCS obsessions | 0.535 | <0.001 | <0.001 | 0.387 | <0.001 | 0.010 |
| Y-BOCS compulsions | 0.483 | <0.001 | <0.001 | 0.297 | 0.007 | 0.032 |
| Psychiatric comorbidity count | 0.409 | <0.001 | <0.001 | 0.253 | 0.024 | 0.089 |
| OCPD trait count | 0.428 | <0.001 | <0.001 | 0.329 | 0.003 | 0.017 |
| HDRS total | 0.041 | 0.717 | 0.861 | -0.056 | 0.620 | 0.746 |
| YMRS total | 0.250 | 0.025 | 0.068 | 0.333 | 0.003 | 0.017 |
| BIS total | 0.204 | 0.069 | 0.140 | 0.042 | 0.712 | 0.791 |
| BIS-Cog | 0.066 | 0.559 | 0.729 | -0.079 | 0.487 | 0.730 |
| BIS-Mot | 0.139 | 0.220 | 0.366 | 0.052 | 0.647 | 0.746 |
| BIS-Plan | 0.211 | 0.061 | 0.140 | 0.055 | 0.625 | 0.746 |
| BPAQ total | 0.077 | 0.500 | 0.681 | -0.057 | 0.616 | 0.746 |
| BPAQ-PA | 0.003 | 0.977 | 0.982 | -0.120 | 0.290 | 0.513 |
| BPAQ-VA | 0.134 | 0.236 | 0.373 | 0.015 | 0.894 | 0.925 |
| BPAQ-A | 0.010 | 0.927 | 0.982 | -0.023 | 0.838 | 0.898 |
| BPAQ-H | 0.041 | 0.716 | 0.861 | -0.125 | 0.270 | 0.506 |
| Contamination/cleaning | -0.204 | 0.070 | 0.140 | -0.213 | 0.058 | 0.194 |
| Taboo thoughts | -0.228 | 0.042 | 0.104 | -0.207 | 0.065 | 0.196 |
| Doubts/checking | -0.006 | 0.955 | 0.982 | -0.066 | 0.561 | 0.746 |
| Symmetry/ordering | 0.003 | 0.982 | 0.982 | -0.004 | 0.971 | 0.971 |
| Hoarding | 0.335 | 0.002 | 0.010 | 0.188 | 0.094 | 0.235 |

*Note.* Benjamini–Hochberg correction was applied separately across all 30 candidate associations for each outcome. Sex, relationship status, employment status, and illness course were coded as indicated in the variable labels. BABS, Brown Assessment of Beliefs Scale; FDR, false discovery rate; OCPD, obsessive–compulsive personality disorder; Y-BOCS, Yale–Brown Obsessive–Compulsive Scale.

Supplementary Table S2. Conditional-network edge estimates and bootstrap stability (N = 80).

**Panel A. Network centred on BABS total**

| Node 1 | Node 2 | Partial r | Selected | Same sign | Bootstrap median | 2.5% | 97.5% |
|---|---|---|---|---|---|---|---|
| BABS total | Age | 0.033 | 52.0% | 51.7% | 0.003 | 0.000 | 0.188 |
| BABS total | Illness duration | 0.015 | 51.8% | 51.7% | 0.006 | 0.000 | 0.184 |
| BABS total | Episodic course | -0.095 | 78.9% | 78.9% | -0.076 | -0.240 | 0.000 |
| BABS total | Y-BOCS total | 0.251 | 99.2% | 99.2% | 0.239 | 0.034 | 0.421 |
| BABS total | Psychiatric comorbidity count | 0.093 | 74.3% | 74.2% | 0.066 | 0.000 | 0.274 |
| BABS total | OCPD traits | 0.117 | 87.2% | 87.2% | 0.104 | 0.000 | 0.307 |
| BABS total | Hoarding | 0.058 | 70.8% | 70.8% | 0.048 | 0.000 | 0.226 |
| BABS total | YMRS total | 0.098 | 75.7% | 75.6% | 0.079 | 0.000 | 0.285 |
| Age | Employment (active=1) | -0.066 | 70.8% | 70.8% | -0.062 | -0.257 | 0.000 |
| Age | Illness duration | 0.368 | 100.0% | 100.0% | 0.351 | 0.147 | 0.552 |
| Age | Psychiatric comorbidity count | 0.188 | 96.8% | 96.8% | 0.165 | 0.000 | 0.322 |
| Employment (active=1) | Illness duration | -0.114 | 87.2% | 87.2% | -0.095 | -0.258 | 0.000 |
| Employment (active=1) | Episodic course | 0.001 | 35.6% | 35.6% | 0.000 | 0.000 | 0.066 |
| Employment (active=1) | Y-BOCS total | -0.195 | 97.3% | 97.3% | -0.178 | -0.343 | 0.000 |
| Employment (active=1) | Psychiatric comorbidity count | -0.169 | 96.5% | 96.5% | -0.155 | -0.311 | 0.000 |
| Illness duration | Episodic course | -0.076 | 71.4% | 71.4% | -0.058 | -0.216 | 0.000 |
| Illness duration | Psychiatric comorbidity count | 0.025 | 58.4% | 58.0% | 0.018 | 0.000 | 0.187 |
| Illness duration | OCPD traits | 0.215 | 98.0% | 98.0% | 0.195 | 0.008 | 0.357 |
| Illness duration | Hoarding | 0.114 | 88.0% | 88.0% | 0.098 | 0.000 | 0.249 |
| Episodic course | Y-BOCS total | -0.112 | 85.7% | 85.7% | -0.101 | -0.260 | 0.000 |
| Episodic course | Psychiatric comorbidity count | -0.001 | 48.3% | 47.5% | 0.000 | -0.153 | 0.000 |
| Episodic course | Hoarding | -0.040 | 70.9% | 70.9% | -0.025 | -0.118 | 0.000 |
| Episodic course | YMRS total | -0.029 | 62.5% | 61.8% | -0.025 | -0.165 | 0.000 |
| Y-BOCS total | Psychiatric comorbidity count | 0.155 | 91.9% | 91.9% | 0.143 | 0.000 | 0.325 |
| Y-BOCS total | OCPD traits | 0.099 | 86.6% | 86.6% | 0.087 | 0.000 | 0.227 |
| Y-BOCS total | Hoarding | 0.090 | 77.0% | 77.0% | 0.075 | 0.000 | 0.252 |
| Y-BOCS total | YMRS total | 0.097 | 86.6% | 86.6% | 0.098 | 0.000 | 0.240 |
| Psychiatric comorbidity count | OCPD traits | 0.050 | 62.7% | 62.5% | 0.031 | 0.000 | 0.231 |
| OCPD traits | Hoarding | 0.188 | 95.3% | 95.3% | 0.184 | 0.000 | 0.371 |
| OCPD traits | YMRS total | 0.139 | 92.8% | 92.8% | 0.130 | 0.000 | 0.300 |

**Panel B. Network centred on BABS item 6**

| Node 1 | Node 2 | Partial r | Selected | Same sign | Bootstrap median | 2.5% | 97.5% |
|---|---|---|---|---|---|---|---|
| BABS item 6 | Age | 0.166 | 93.3% | 93.3% | 0.139 | 0.000 | 0.319 |
| BABS item 6 | Illness duration | 0.010 | 54.3% | 54.0% | 0.008 | 0.000 | 0.171 |
| BABS item 6 | Y-BOCS total | 0.140 | 88.7% | 88.7% | 0.119 | 0.000 | 0.296 |
| BABS item 6 | OCPD traits | 0.046 | 62.9% | 62.8% | 0.031 | 0.000 | 0.244 |
| BABS item 6 | YMRS total | 0.198 | 95.2% | 95.2% | 0.186 | 0.000 | 0.380 |

| Node 1 | Node 2 | Partial r | Selected | Same sign | Bootstrap median | 2.5% | 97.5% |
|---|---|---|---|---|---|---|---|
| Age | Employment (active=1) | -0.063 | 71.2% | 71.0% | -0.058 | -0.277 | 0.000 |
| Age | Illness duration | 0.368 | 100.0% | 100.0% | 0.352 | 0.144 | 0.536 |
| Age | Psychiatric comorbidity count | 0.191 | 97.4% | 97.4% | 0.164 | 0.000 | 0.333 |
| Employment (active=1) | Illness duration | -0.118 | 84.6% | 84.6% | -0.097 | -0.269 | 0.000 |
| Employment (active=1) | Episodic course | 0.006 | 49.8% | 49.8% | 0.000 | 0.000 | 0.083 |
| Employment (active=1) | Y-BOCS total | -0.203 | 98.3% | 98.3% | -0.190 | -0.357 | -0.021 |
| Employment (active=1) | Psychiatric comorbidity count | -0.177 | 96.3% | 96.3% | -0.176 | -0.342 | 0.000 |
| Illness duration | Episodic course | -0.085 | 73.8% | 73.8% | -0.067 | -0.222 | 0.000 |
| Illness duration | Psychiatric comorbidity count | 0.027 | 61.7% | 61.4% | 0.027 | 0.000 | 0.189 |
| Illness duration | OCPD traits | 0.219 | 97.3% | 97.3% | 0.195 | 0.000 | 0.381 |
| Illness duration | Hoarding | 0.121 | 90.9% | 90.9% | 0.101 | 0.000 | 0.250 |
| Episodic course | Y-BOCS total | -0.145 | 92.2% | 92.2% | -0.124 | -0.265 | 0.000 |
| Episodic course | Psychiatric comorbidity count | -0.017 | 52.4% | 51.9% | -0.006 | -0.169 | 0.000 |
| Episodic course | Hoarding | -0.056 | 81.5% | 81.5% | -0.043 | -0.139 | 0.000 |
| Episodic course | YMRS total | -0.047 | 71.1% | 70.3% | -0.048 | -0.189 | 0.000 |
| Y-BOCS total | Psychiatric comorbidity count | 0.184 | 95.7% | 95.6% | 0.164 | 0.000 | 0.345 |
| Y-BOCS total | OCPD traits | 0.126 | 91.9% | 91.9% | 0.116 | 0.000 | 0.274 |
| Y-BOCS total | Hoarding | 0.113 | 86.4% | 86.3% | 0.101 | 0.000 | 0.270 |
| Y-BOCS total | YMRS total | 0.100 | 90.2% | 90.2% | 0.103 | 0.000 | 0.254 |
| Psychiatric comorbidity count | OCPD traits | 0.066 | 69.3% | 69.3% | 0.054 | 0.000 | 0.241 |
| OCPD traits | Hoarding | 0.205 | 96.6% | 96.6% | 0.195 | 0.000 | 0.387 |
| OCPD traits | YMRS total | 0.147 | 94.8% | 94.8% | 0.141 | 0.000 | 0.320 |

*Note.* Partial r values are full-sample conditional associations estimated by graphical lasso. Selected is the percentage of 1,000 bootstrap samples in which the edge was retained; Same sign is the percentage preserving the full-sample direction. Bootstrap intervals are percentile intervals and may include zero because the regularized estimator sets non-selected edges to zero.

Supplementary Table S3. Bootstrap stability of relative-importance estimates and rankings (N = 80).

**Panel A. BABS total ($R^2$ = 0.400; estimable resamples = 1,000/1,000)**

| Clinical variable | Point estimate (%) | Bootstrap median (%) | 2.5% | 97.5% | Rank 1 (%) | Top 2 (%) |
|---|---|---|---|---|---|---|
| Y-BOCS total | 37.2 | 32.2 | 11.2 | 59.7 | 66.1 | 86.7 |
| OCPD trait count | 13.6 | 13.0 | 2.8 | 34.0 | 9.3 | 29.8 |
| YMRS total | 18.6 | 15.3 | 1.9 | 40.9 | 14.9 | 43.1 |
| Age | 5.6 | 5.4 | 1.0 | 19.5 | 0.3 | 6.0 |
| Illness duration | 6.3 | 6.5 | 1.2 | 22.3 | 1.8 | 5.9 |
| Psychiatric comorbidity count | 11.6 | 10.8 | 2.5 | 37.0 | 7.2 | 24.9 |
| Hoarding | 7.1 | 6.8 | 1.3 | 18.7 | 0.4 | 3.6 |

**Panel B. BABS item 6 >0 (McFadden pseudo-$R^2$ = 0.185; n above threshold = 64; estimable resamples = 1,000/1,000)**

| Clinical variable | Point estimate (%) | Bootstrap median (%) | 2.5% | 97.5% | Rank 1 (%) | Top 2 (%) |
|---|---|---|---|---|---|---|
| Y-BOCS total | 5.3 | 5.8 | 0.9 | 30.5 | 4.0 | 10.3 |
| OCPD trait count | 4.8 | 5.4 | 1.3 | 22.0 | 1.0 | 5.2 |
| YMRS total | 10.7 | 9.4 | 0.7 | 42.9 | 9.8 | 22.1 |
| Age | 40.3 | 30.4 | 4.4 | 60.0 | 51.1 | 74.7 |
| Illness duration | 25.6 | 20.1 | 2.9 | 50.3 | 20.7 | 57.9 |
| Psychiatric comorbidity count | 9.1 | 7.7 | 1.3 | 39.4 | 9.8 | 20.4 |
| Hoarding | 4.4 | 4.6 | 0.8 | 31.4 | 3.6 | 9.4 |

**Panel C. BABS item 6 >1 (McFadden pseudo-$R^2$ = 0.217; n above threshold = 42; estimable resamples = 1,000/1,000)**

| Clinical variable | Point estimate (%) | Bootstrap median (%) | 2.5% | 97.5% | Rank 1 (%) | Top 2 (%) |
|---|---|---|---|---|---|---|
| Y-BOCS total | 24.7 | 19.2 | 4.3 | 48.9 | 27.2 | 52.2 |
| OCPD trait count | 28.9 | 23.5 | 5.0 | 54.0 | 41.2 | 64.7 |
| YMRS total | 14.2 | 12.2 | 1.2 | 37.2 | 11.6 | 29.0 |
| Age | 12.3 | 10.1 | 1.3 | 37.8 | 10.0 | 24.1 |
| Illness duration | 5.8 | 6.2 | 1.9 | 21.6 | 1.2 | 5.0 |
| Psychiatric comorbidity count | 6.7 | 6.6 | 1.6 | 29.2 | 4.6 | 12.1 |
| Hoarding | 7.4 | 7.2 | 1.6 | 29.0 | 4.2 | 12.9 |

**Panel D. BABS item 6 >2 (McFadden pseudo-$R^2$ = 0.323; n above threshold = 11; estimable resamples = 1,000/1,000)**

| Clinical variable | Point estimate (%) | Bootstrap median (%) | 2.5% | 97.5% | Rank 1 (%) | Top 2 (%) |
|---|---|---|---|---|---|---|
| Y-BOCS total | 11.2 | 10.2 | 1.3 | 37.1 | 6.6 | 29.6 |
| OCPD trait count | 2.7 | 4.1 | 0.8 | 23.0 | 1.1 | 8.5 |
| YMRS total | 53.8 | 39.2 | 4.3 | 73.0 | 70.9 | 85.5 |
| Age | 20.0 | 16.3 | 2.0 | 44.6 | 15.7 | 47.7 |
| Illness duration | 3.8 | 5.2 | 0.8 | 21.6 | 0.6 | 6.0 |
| Psychiatric comorbidity count | 6.0 | 7.1 | 1.1 | 34.5 | 4.7 | 18.4 |
| Hoarding | 2.3 | 4.3 | 1.0 | 18.9 | 0.4 | 4.3 |

*Note.* Relative-importance values are percentages of the within-model fit measure. Bootstrap stability was assessed using 1,000 nonparametric resamples with fixed seed 20260811. Percentile intervals and rank frequencies were calculated across all 1,000 resamples for each primary model; all

primary resamples were estimable. The >3 contrast was not included in the primary relative-importance analysis because only four participants exceeded this threshold.

### Supplementary Table S4. Conventional multivariable and planned sensitivity analyses.

#### Panel A. Conventional fully adjusted BABS total model

| Variable | Standardized β | 95% CI lower | 95% CI upper | p |
|---|---|---|---|---|
| Y-BOCS total | 0.339 | 0.051 | 0.628 | 0.021 |
| OCPD trait count | 0.100 | -0.161 | 0.362 | 0.453 |
| YMRS total | 0.222 | -0.071 | 0.515 | 0.138 |
| Age | 0.081 | -0.200 | 0.362 | 0.572 |
| Illness duration | 0.052 | -0.203 | 0.307 | 0.689 |
| Psychiatric comorbidity count | 0.100 | -0.242 | 0.441 | 0.566 |
| Hoarding | 0.065 | -0.115 | 0.245 | 0.480 |

Model fit: $R^2 = 0.400$; adjusted $R^2 = 0.342$; global $p < 0.001$.

#### Panel B. Conventional partial proportional-odds model for BABS item 6

| Variable or cumulative contrast | OR per 1-SD increase | 95% CI lower | 95% CI upper | p |
|---|---|---|---|---|
| Y-BOCS total | 1.588 | 0.920 | 2.741 | 0.097 |
| OCPD trait count | 1.370 | 0.792 | 2.369 | 0.260 |
| Age | 1.975 | 1.115 | 3.499 | 0.020 |
| Illness duration | 1.127 | 0.643 | 1.975 | 0.676 |
| Psychiatric comorbidity count | 0.859 | 0.498 | 1.481 | 0.585 |
| Hoarding | 0.885 | 0.547 | 1.430 | 0.617 |
| YMRS total: Y > 0 | 1.329 | 0.572 | 3.088 | 0.509 |
| YMRS total: Y > 1 | 1.619 | 0.791 | 3.316 | 0.187 |
| YMRS total: Y > 2 | 3.021 | 1.452 | 6.286 | 0.003 |
| YMRS total: Y > 3 | 2.622 | 1.285 | 5.350 | 0.008 |

Model fit: LR $\chi^2(10) = 33.968$; global $p < 0.001$; McFadden pseudo-$R^2 = 0.151$.

#### Panel C. Selected estimates from prespecified sensitivity analyses

| Analysis | Outcome | Variable | Estimate | 95% CI lower | 95% CI upper | p |
|---|---|---|---|---|---|---|
| decomp | BABS total | Y-BOCS obsessions | 0.288 | -0.133 | 0.710 | 0.180 |
| decomp | BABS total | Y-BOCS compulsions | 0.065 | -0.394 | 0.524 | 0.781 |
| decomp | BABS total | YMRS total | 0.222 | -0.067 | 0.512 | 0.132 |
| decomp | BABS total | Age | 0.076 | -0.212 | 0.363 | 0.605 |
| decomp | BABS item 6 (proportional odds) | Y-BOCS obsessions | 2.106 | 0.787 | 5.637 | 0.138 |
| decomp | BABS item 6 (proportional odds) | Y-BOCS compulsions | 0.717 | 0.264 | 1.946 | 0.514 |
| decomp | BABS item 6 (proportional odds) | YMRS total | 2.250 | 1.254 | 4.036 | 0.007 |
| decomp | BABS item 6 (proportional odds) | Age | 1.867 | 1.034 | 3.370 | 0.038 |
| extended | BABS total | Y-BOCS total | 0.313 | -0.006 | 0.631 | 0.054 |
| extended | BABS total | YMRS total | 0.216 | -0.090 | 0.523 | 0.166 |
| extended | BABS total | Age | 0.081 | -0.213 | 0.374 | 0.591 |
| extended | BABS total | Employment (active=1) | -0.007 | -0.243 | 0.229 | 0.953 |
| extended | BABS total | Episodic course | -0.093 | -0.297 | 0.112 | 0.375 |
| extended | BABS item 6 (proportional odds) | Y-BOCS total | 1.607 | 0.752 | 3.432 | 0.221 |

| Analysis | Outcome | Variable | Estimate | 95% CI lower | 95% CI upper | p |
|---|---|---|---|---|---|---|
| extended | BABS item 6 (proportional odds) | YMRS total | 2.207 | 1.215 | 4.007 | 0.009 |
| extended | BABS item 6 (proportional odds) | Age | 1.951 | 0.977 | 3.896 | 0.058 |
| extended | BABS item 6 (proportional odds) | Employment (active=1) | 1.119 | 0.612 | 2.046 | 0.716 |
| extended | BABS item 6 (proportional odds) | Episodic course | 1.035 | 0.762 | 1.407 | 0.825 |
| taboo | BABS total | Y-BOCS total | 0.365 | 0.044 | 0.685 | 0.026 |
| taboo | BABS total | YMRS total | 0.209 | -0.071 | 0.490 | 0.144 |
| taboo | BABS total | Age | 0.085 | -0.191 | 0.360 | 0.546 |
| taboo | BABS total | Taboo thoughts | -0.134 | -0.374 | 0.106 | 0.274 |
| taboo | BABS item 6 (proportional odds) | Y-BOCS total | 1.664 | 0.831 | 3.332 | 0.150 |
| taboo | BABS item 6 (proportional odds) | YMRS total | 2.154 | 1.172 | 3.958 | 0.013 |
| taboo | BABS item 6 (proportional odds) | Age | 1.933 | 1.073 | 3.482 | 0.028 |
| taboo | BABS item 6 (proportional odds) | Taboo thoughts | 0.751 | 0.461 | 1.225 | 0.252 |
| sex | BABS total | Y-BOCS total | 0.338 | 0.043 | 0.633 | 0.025 |
| sex | BABS total | YMRS total | 0.243 | -0.049 | 0.535 | 0.103 |
| sex | BABS total | Age | 0.083 | -0.197 | 0.362 | 0.561 |
| sex | BABS total | Sex (male=1) | -0.103 | -0.332 | 0.126 | 0.379 |
| sex | BABS item 6 (proportional odds) | Y-BOCS total | 1.521 | 0.814 | 2.843 | 0.189 |
| sex | BABS item 6 (proportional odds) | YMRS total | 2.263 | 1.251 | 4.093 | 0.007 |
| sex | BABS item 6 (proportional odds) | Age | 1.894 | 1.044 | 3.439 | 0.036 |
| sex | BABS item 6 (proportional odds) | Sex (male=1) | 0.899 | 0.565 | 1.431 | 0.655 |
| BABS total excluding item 6 | BABS items 1-5 | Y-BOCS total | 0.355 | 0.073 | 0.638 | 0.014 |
| BABS total excluding item 6 | BABS items 1-5 | OCPD trait count | 0.106 | -0.154 | 0.365 | 0.424 |
| BABS total excluding item 6 | BABS items 1-5 | YMRS total | 0.183 | -0.109 | 0.476 | 0.220 |
| BABS total excluding item 6 | BABS items 1-5 | Age | 0.027 | -0.254 | 0.309 | 0.850 |
| BABS total excluding item 6 | BABS items 1-5 | Illness duration | 0.055 | -0.210 | 0.320 | 0.684 |
| BABS total excluding item 6 | BABS items 1-5 | Psychiatric comorbidity count | 0.133 | -0.201 | 0.466 | 0.435 |
| BABS total excluding item 6 | BABS items 1-5 | Hoarding | 0.093 | -0.087 | 0.273 | 0.314 |
| Four-category partial proportional odds | BABS item 6 | Y-BOCS total | 1.607 | 0.930 | 2.776 | 0.089 |
| Four-category partial proportional odds | BABS item 6 | OCPD trait count | 1.424 | 0.823 | 2.466 | 0.206 |
| Four-category partial proportional odds | BABS item 6 | Age | 2.040 | 1.142 | 3.645 | 0.016 |
| Four-category partial proportional odds | BABS item 6 | Illness duration | 1.123 | 0.642 | 1.963 | 0.685 |
| Four-category partial proportional odds | BABS item 6 | Psychiatric comorbidity count | 0.850 | 0.493 | 1.466 | 0.559 |
| Four-category partial proportional odds | BABS item 6 | Hoarding | 0.867 | 0.532 | 1.411 | 0.565 |
| Four-category partial proportional odds | BABS item 6 | YMRS total: >0 | 1.281 | 0.554 | 2.963 | 0.562 |

| Analysis | Outcome | Variable | Estimate | 95% CI lower | 95% CI upper | p |
|---|---|---|---|---|---|---|
| Four-category partial proportional odds | BABS item 6 | YMRS total: >1 | 1.559 | 0.766 | 3.173 | 0.221 |
| Four-category partial proportional odds | BABS item 6 | YMRS total: >2 | 2.738 | 1.403 | 5.341 | 0.003 |
| Ridge CV | BABS total | Y-BOCS total | 0.222 | | | |
| Ridge CV | BABS total | OCPD trait count | 0.104 | | | |
| Ridge CV | BABS total | YMRS total | 0.157 | | | |
| Ridge CV | BABS total | Age | 0.064 | | | |
| Ridge CV | BABS total | Illness duration | 0.060 | | | |
| Ridge CV | BABS total | Psychiatric comorbidity count | 0.101 | | | |
| Ridge CV | BABS total | Hoarding | 0.072 | | | |
| Elastic net CV | BABS total | Y-BOCS total | 0.257 | | | |
| Elastic net CV | BABS total | OCPD trait count | 0.101 | | | |
| Elastic net CV | BABS total | YMRS total | 0.165 | | | |
| Elastic net CV | BABS total | Age | 0.057 | | | |
| Elastic net CV | BABS total | Illness duration | 0.050 | | | |
| Elastic net CV | BABS total | Psychiatric comorbidity count | 0.094 | | | |
| Elastic net CV | BABS total | Hoarding | 0.059 | | | |

*Note.* For BABS total sensitivity models, unpenalized estimates are standardized β coefficients. For BABS item 6 sensitivity models, unpenalized estimates are odds ratios. Ridge and elastic-net estimates are standardized penalized coefficients and are presented as coefficient-stability checks; confidence intervals and p values are not applicable to these penalized estimates. Complete prespecified sensitivity outputs and diagnostic results are provided in Supplementary Data S2.

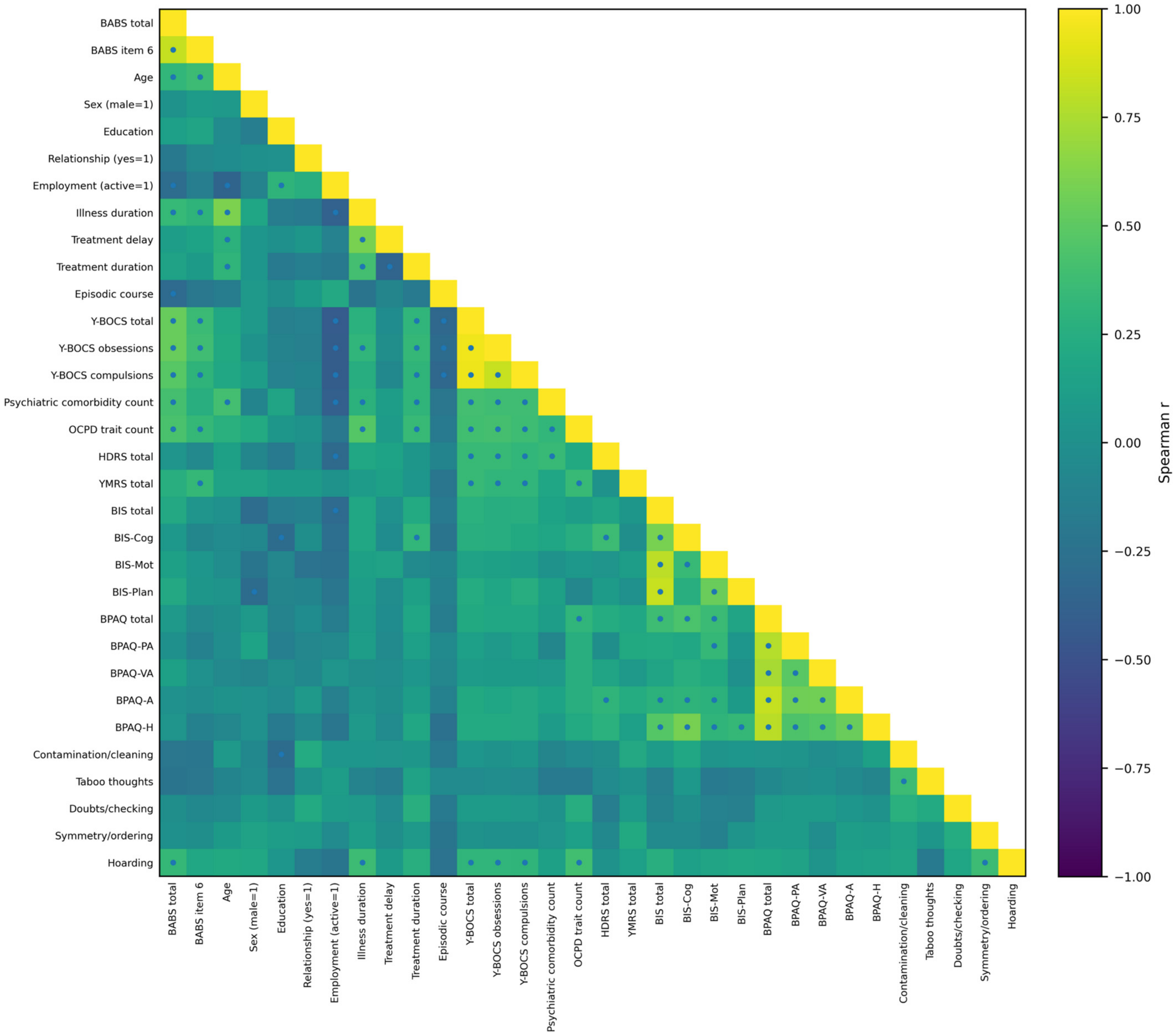


Supplementary Figure S1. Full Spearman association matrix of insight measures and clinical variables (N = 80).

*Note.* The lower triangle displays the pairwise Spearman correlation structure for all 32 variables, including BABS total and BABS item 6. Dots identify associations remaining significant at q < 0.05 after Benjamini–Hochberg correction across all 496 unique pairwise associations. Complete r, p, and q matrices and coding notes are provided in Supplementary Data S1.
