# Supplementary material for: Clinical Correlates of Poor Insight in Obsessive–Compulsive Disorder: An Integrative Cross-Sectional Analysis
Source: J Clin Med. 2026 Sep 19;15(18):7289. doi: 10.3390/jcm15187289 (PMC13607875; doi:10.3390/jcm15187289)
Supplement: Supplementary file 1 [file jcm-15-07289-s001.zip › Supplementary Material S2.pdf]

Supplementary Material S2

Clinical Correlates of Poor Insight in Obsessive–Compulsive Disorder: An Integrative Cross-Sectional Analysis

Diagnostic Verification Form

English version

Purpose and use. This form documents the clinician-led diagnostic verification procedure used in the study for the primary diagnosis of obsessive–compulsive disorder (OCD), selected psychiatric comorbidity categories, obsessive–compulsive personality disorder (OCPD) traits, and illness-history variables reported in the manuscript. Verification was based on clinical assessment and available psychiatric records and was guided by DSM-5 diagnostic criteria where applicable. The form served as a structured aid to clinician-led verification and was not used as a stand-alone diagnostic instrument.

Scoring framework. The psychiatric comorbidity count comprises four categories: major depressive disorder, anxiety disorders, bipolar disorder, and alcohol dependence/abuse. Each category contributes 0 or 1 point to the count (possible range 0–4; observed range in the study 0–3). OCPD was assessed separately and analyzed dimensionally as the number of DSM-5 OCPD criteria present (0–8).

Study ID		Date of assessment	
Clinician		Source reviewed	[ ] Clinical interview [ ] Psychiatric records [ ] Both

A. Primary obsessive–compulsive disorder and illness history

Primary OCD diagnosis: [ ] DSM-5 criteria fulfilled [ ] diagnosis documented in psychiatric records

Independent clinical confirmation by at least two experienced clinicians: [ ] Confirmed [ ] Not confirmed

OCD diagnostic verification considered the presence of obsessions, compulsions, or both; symptoms that were time-consuming (typically >1 h/day) or caused clinically significant distress or impairment; exclusion of substance, medication, or medical causes; and exclusion of a better explanation by another mental disorder.

**Age at onset of OCD symptoms**	______________________________
**Illness duration**	______________________________ years
**Treatment delay**	______________________________ years
**Treatment duration**	______________________________ years
**Illness course**	[ ] Chronic [ ] Episodic
**Course definition**	Episodic: at least one remission lasting ≥12 months. Chronic: no remission lasting ≥12 months.

Operational definitions used in the manuscript: illness duration = interval from OCD symptom onset to study assessment; treatment delay = interval from symptom onset to initiation of OCD treatment; treatment duration = interval from treatment initiation to study assessment.

B. Psychiatric comorbidity verification

For each category, record the diagnostic conclusion used for the study and the basis for verification. A category contributes one point to the psychiatric comorbidity count if present.

B1. Major depressive disorder

- History consistent with a major depressive episode lasting at least 2 weeks, with the required symptom pattern (including depressed mood and/or loss of interest or pleasure) and clinically significant distress or functional impairment.
- Symptoms not better attributable to a substance, medication, or medical condition; schizophrenia-spectrum or other psychotic disorders were considered where clinically relevant.
- No history meeting criteria for a manic or hypomanic episode; bipolar disorder was considered in the differential diagnosis.

**Diagnostic status**	[ ] Present [ ] Absent [ ] Uncertain
**Documented in psychiatric records**	[ ] Yes [ ] No
**Verification note / relevant diagnosis**	____

B2. Anxiety disorders

- Generalized anxiety disorder: excessive anxiety/worry about several events, occurring more days than not for at least 6 months, difficult to control, with associated symptoms (at least three in adults), clinically significant distress or impairment, and appropriate diagnostic exclusions.
- Panic disorder: recurrent unexpected panic attacks, with at least one attack followed by at least 1 month of persistent concern about additional attacks or their consequences and/or maladaptive behavioral change, with appropriate diagnostic exclusions.

Specific disorder recorded: [ ] Generalized anxiety disorder [ ] Panic disorder [ ] Both

**Diagnostic status**	[ ] Present [ ] Absent [ ] Uncertain
**Documented in psychiatric records**	[ ] Yes [ ] No
**Verification note / relevant diagnosis**	____________________

B3. Bipolar disorder

- Bipolar I disorder: history of a manic episode with the required symptom pattern and duration (at least 1 week, or any duration if hospitalization was required), with marked functional impairment, hospitalization, or psychotic features as clinically applicable.
- Bipolar II disorder: history of a hypomanic episode lasting at least 4 consecutive days together with a major depressive episode, with no lifetime manic episode. Episode-related change from baseline, functional consequences, and exclusion of substance, medication, or medical causes were considered.

Subtype / episode history: [ ] Bipolar I [ ] Bipolar II [ ] Manic episode [ ] Hypomanic episode

**Diagnostic status**	[ ] Present [ ] Absent [ ] Uncertain
**Documented in psychiatric records**	[ ] Yes [ ] No
**Verification note / relevant diagnosis**	____________________

B4. Alcohol dependence/abuse

- Study category: clinically significant problematic alcohol use or a documented diagnosis of alcohol abuse/dependence in the clinical assessment and/or psychiatric records.
- Verification considered impaired control, continued use despite harm, role impairment, hazardous use, tolerance, withdrawal, and other relevant alcohol-use features. This study category reflected clinical documentation and study coding and was not treated as a DSM-5 diagnostic label.

**Diagnostic status**	[ ] Present [ ] Absent [ ] Uncertain
**Documented in psychiatric records**	[ ] Yes [ ] No
**Verification note / relevant diagnosis**	____________________

B5. Psychiatric comorbidity count

Category	Present (0/1)	Notes
Major depressive disorder		
Anxiety disorders		
Bipolar disorder		
Alcohol dependence/abuse		
**Psychiatric comorbidity count**	**____ / 4**	**Observed study range: 0–3**

C. Obsessive–compulsive personality disorder traits

OCPD was assessed separately from psychiatric comorbidity with reference to the eight DSM-5 OCPD criteria. Clinician assessment considered whether the pattern was longstanding and pervasive across contexts. Each criterion was recorded as present or absent and summed (0–8); the dimensional OCPD trait count was the variable analyzed in the study.

No.	OCPD trait criterion (paraphrased)	Present	Absent
1	Preoccupation with details, rules, lists, order, organization, or schedules to the extent that the major point of an activity may be lost.	[ ]	[ ]
2	Perfectionism that interferes with task completion because of overly strict standards.	[ ]	[ ]

No.	OCPD trait criterion (paraphrased)	Present	Absent
3	Excessive devotion to work and productivity at the expense of leisure activities and relationships, beyond economic necessity.	[ ]	[ ]
4	Over-conscientiousness, scrupulousness, and inflexibility regarding matters of morality, ethics, or values beyond cultural or religious context.	[ ]	[ ]
5	Difficulty discarding worn-out or worthless objects even when they have no sentimental value.	[ ]	[ ]
6	Reluctance to delegate tasks or work with others unless they agree to do things in the person's way.	[ ]	[ ]
7	A miserly spending style toward self and others, with money viewed as something to be accumulated for future catastrophes.	[ ]	[ ]
8	Rigidity and stubbornness.	[ ]	[ ]
**OCPD trait count (number of criteria present)**		**______ / 8**	

D. Final verification

**Clinician completing the form**	____
**Date**	____
**Primary OCD diagnosis independently confirmed by second experienced clinician**	[ ] Yes [ ] No
**Second clinician / verification date**	____

Note. *This blank English-language version of the clinician-completed diagnostic verification form used in the study is provided as supplementary documentation. It contains no participant-identifiable information.*
